# A geometric calibration method for the digital chest tomosynthesis with dual-axis scanning geometry

**DOI:** 10.1371/journal.pone.0216054

**Published:** 2019-04-25

**Authors:** Chia-Hao Chang, Yu-Ching Ni, Syuan-Ya Huang, Ho-Hui Hsieh, Sheng-Pin Tseng, Fan-Pin Tseng

**Affiliations:** Health Physics Division, Institute of Nuclear Energy Research, Atomic Energy Council, Taoyuan, Taiwan; Stanford University School of Medicine, UNITED STATES

## Abstract

The aim of this study was to develop a geometric calibration method capable of eliminating the reconstruction artifacts of geometric misalignments in a tomosynthesis prototype with dual-axis scanning geometry. The potential scenarios of geometric misalignments were demonstrated, and their effects on reconstructed images were also evaluated. This method was a phantom-based approach with iterative optimization, and the calibration phantom was designed for specific tomosynthesis scanning geometry. The phantom was used to calculate a set of geometric parameters from each projection, and these parameters were then used to evaluate the geometric misalignments of the dual-axis scanning-geometry prototype. The simulated results revealed that the extracted geometric parameters were similar to the input values and that the artifacts of reconstructed images were minimized due to geometric calibration. Additionally, experimental chest phantom imaging results also indicated that the artifacts of the reconstructed images were suppressed and that object structures were preserved through calibration. And the quantitative analysis result also indicated that the MTF can be further improved with the geometric calibration. All the simulated and experimental results demonstrated that this method is effective for tomosynthesis with dual-axis scanning geometry. Furthermore, this geometric calibration method can also be applied to other tomography imaging systems to reduce geometric misalignments and be used for different geometric calibration phantom configurations.

## Introduction

**Fig 1:**
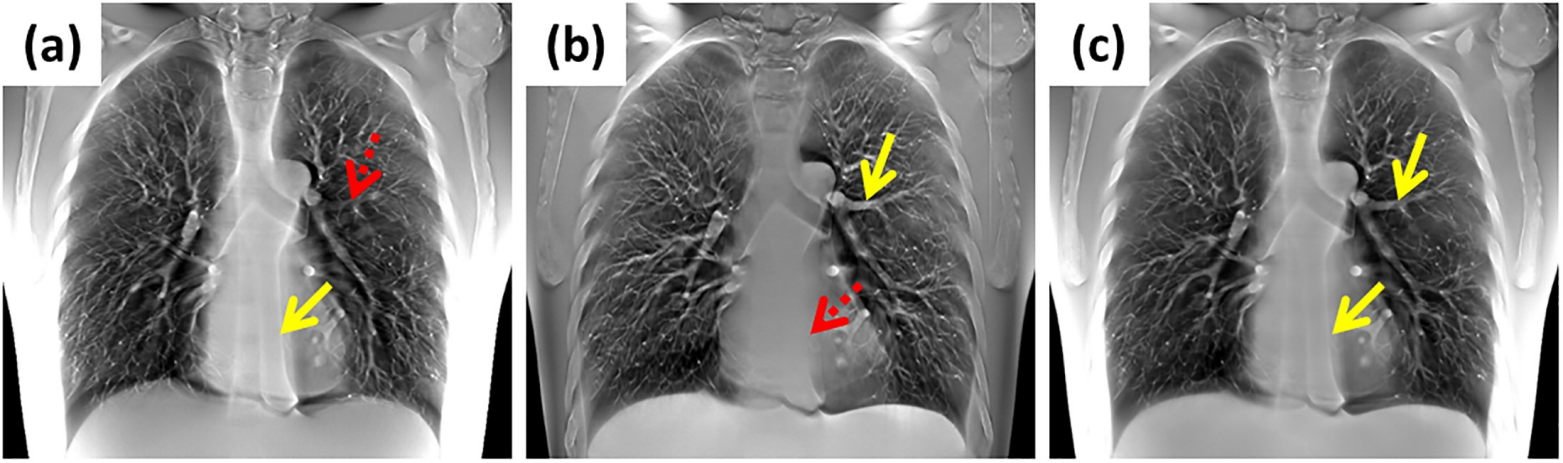
Reconstructed images of a digital chest tomosynthesis system with different X-ray tube sweep directions. (a) Left–right (LR)-axis scanning geometry. (b) Head–foot (HF)-axis scanning geometry. (c) Dual-axis scanning geometry. An anthropomorphic chest phantom (Model: LUNGMAN, Kyoto Kagaku, Japan) was used to demonstrate differences between single-axis and dual-axis scanning geometry.

Traditional digital tomosynthesis (DTS) systems have been commonly used as clinical diagnostic tools in various medical imaging applications such as digital breast tomosynthesis and digital chest tomosynthesis [1–4]. During DTS imaging, a series of projection images of objects are acquired at a limited angle. These systems can provide images of computed tomography (CT)-like quality with in-depth information and at lower dose of radiation [1]. However, the image quality of traditional chest DTS is limited because it only uses single-axis scanning geometry, as indicated in Fig 1a and 1b. Some structures parallel to the scanning direction are blurred (red arrows) because of ghost artifact distortion [5]. Most chest DTS systems used in clinical settings have only the head–foot (HF)-axis scanning direction, resulting in the overlapping of the spinal and thoracic–aorta area (red arrows). Other studies have indicated that expanding the area of the scanning coverage can be more effective than increasing the sampling density for improving tomosynthesis image quality [6, 7]. Hence, as shown in Fig 1c, these limitations can be partially overcome by using dual-axis scanning geometry with a larger scanning coverage area (yellow arrows) [7, 8]. Nevertheless, the requirement for geometry alignment is higher for the system with dual-axis scanning geometry than for traditional tomosynthesis with single-axis scanning geometry. Simulation studies have demonstrated that image artifacts are induced by the geometric misalignments of the system, including axis shift and tilt. For such systems, the positions of the two scanning axes and image receptor are difficult to line up with the mechanical alignment method. Hence, a geometric calibration method must be used to obtain high-precision geometric parameters of the tomosynthesis system with dual-axis scanning geometry.

The geometric calibration method can be classified into phantom-based methods [9–20] and phantom-free methods [21–24] based on whether the calibration phantom is applied in the geometric calibration process. For phantom-based methods, a calibration phantom consisting of numerous markers is used to extract geometric parameters in each projection view. A phantom-based method can be used to calculate geometric parameters by mapping the relationship between the projected positions and predefined coordinates of markers. The calculation accuracy of phantom-based methods crucially depends on the manufacturing precision of the calibration phantom [25]. For phantom-free methods, geometric parameters are extracted without using the calibration phantom. Phantom-free methods can be used to calculate geometric parameters from projection images by minimizing the objective function. For the phantom-free method, it requires a multitude of projections to achieve sufficient accuracy for geometric parameters [24]. Hence, the phantom-based method is more suitable for tomosynthesis with limited angle-scanning geometry.

In this study, a phantom-based calibration method was developed to calibrate the geometric misalignments of the tomosynthesis system with dual-axis scanning geometry. The method was a projection matrix-based approach that involved an iterative minimization process, which can be used to reduce the number of errors in projection matrix calculations. By using the calibration phantom, a set of geometric parameters were extracted from each projection view and subsequently used in tomosynthesis reconstruction to eliminate artifacts resulting from geometric misalignments.

This paper is organized as follows. The materials and methods section provides a description of the imaging system, scenarios of geometric misalignments in the dual-axis scanning geometry system, mathematical foundation of the geometric calibration method, and calibration of the phantom design. This method was used to calculate the geometric parameters of the tomosynthesis system with dual-axis scanning geometry, and the results section presents an evaluation of the calculation accuracy of extracted geometric parameters. To further assess the effects of geometric misalignments, reconstructed images were simulated for various scenarios. Moreover, tomosynthesis experiments were implemented using a chest phantom to validate the calibration performance, and reconstructed images with and without geometric calibration were compared. In the final section, the major findings of this study are discussed and summarized.

## Materials and methods

### Digital chest tomosynthesis prototype with dual-axis scanning geometry

**Fig 2:**
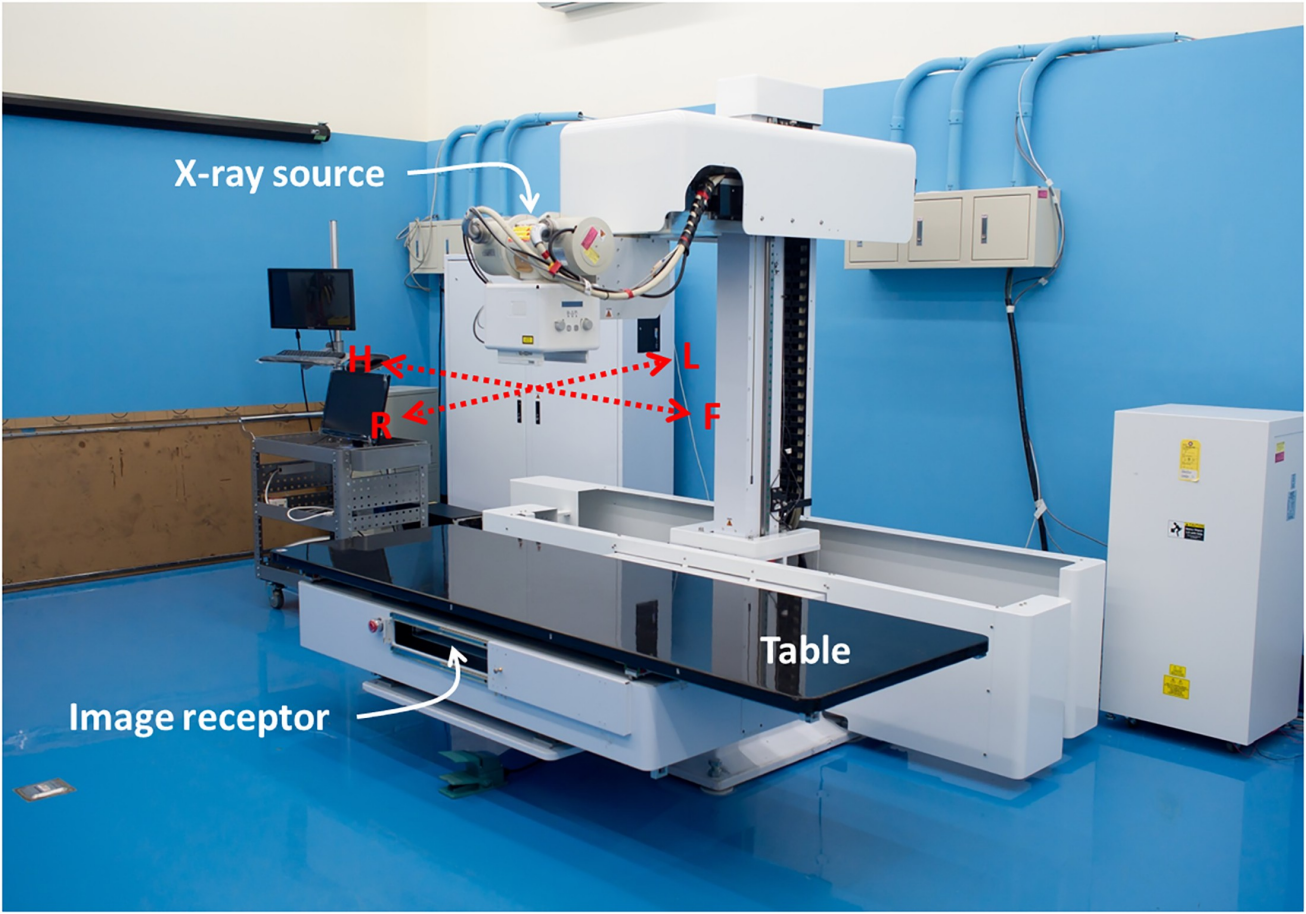
TomoDR prototype. TomoDR has dual-axis scanning geometry (HF-axis and LR-axis). Compare to the traditional single-axis tomosynthesis, TomoDR can provide more information in the reconstructed images.

TomoDR is a self-developed tomosynthesis prototype equipped with dual-axis scanning geometry in two perpendicular directions (Fig 2). [26] This system was assembled with a medical X-ray source (Model: SG-1096, Varian Medical Systems, USA) and a digital flat-panel image receptor (Model: PaxScan 4343CB, Varian Medical Systems, USA). The motion mechanism of the dual axis can provide a position with an accuracy of 50 μm, which can meet all the requirements of position repeatability for the digital chest tomosynthesis system.

For system configurations in simulation and experimental studies, the source-to-image-receptor distance (SID) was 1120 mm, and the distance between the rotation isocenter and the image receptor surface was 20 mm. During the process of acquiring projection images, both the object and image receptor were stationary, and only the X-ray source moved along the head–foot (HF) axis or left–right (LR) axis. The image receptor was a 3072 × 3072 pixels matrix flat-panel detector with a pixel size of 0.139 × 0.139 mm^2^. We used binning mode 2 of the image receptor to reduce the amount of time required for the X-ray projection simulation and image reconstruction process. For the clinical chest scanning protocol, the X-ray source position moves from −300 mm (−15.26°) to +300 mm (+15.26°) at 10-mm increments in the HF-axis scanning direction. In the LR-axis scanning direction, the X-ray source position moves from −150 mm (−7.77°) to +150 mm (+7.77°) at 10-mm increments. Hence, the number of HF- and LR-axis projection images was 61 and 31, respectively. For the clinical chest reconstruction image protocol, we reconstructed 92 projection images by using a simultaneous algebraic reconstruction technique (SART) [27] in a 1024 × 1024 × 40 pixels matrix with a voxel size of 0.5 × 0.5 × 5 mm³.

### Scenarios of geometric misalignments of the tomosynthesis with dual-axis scanning geometry

For the DTS with dual-axis scanning geometry, the situation of geometric misalignments is more complex than in the case of single-axis scanning geometry. For single-axis scanning geometry, only one axis movement occurs during the scanning process. Even when the system has an axis shift in single-axis scanning geometry, the influence of the reconstructed image is an object shift without artifacts. Only one axis is required to be aligned with reference axes. However, for the system with dual-axis scanning geometry, the center points of two scanning axes must be coaligned to the same position. Furthermore, the precise angle of tilt of two axes with reference axes should also be known for geometric calibration. Therefore, any misalignments of the two axes lead to artifacts on reconstructed images.

**Fig 3:**
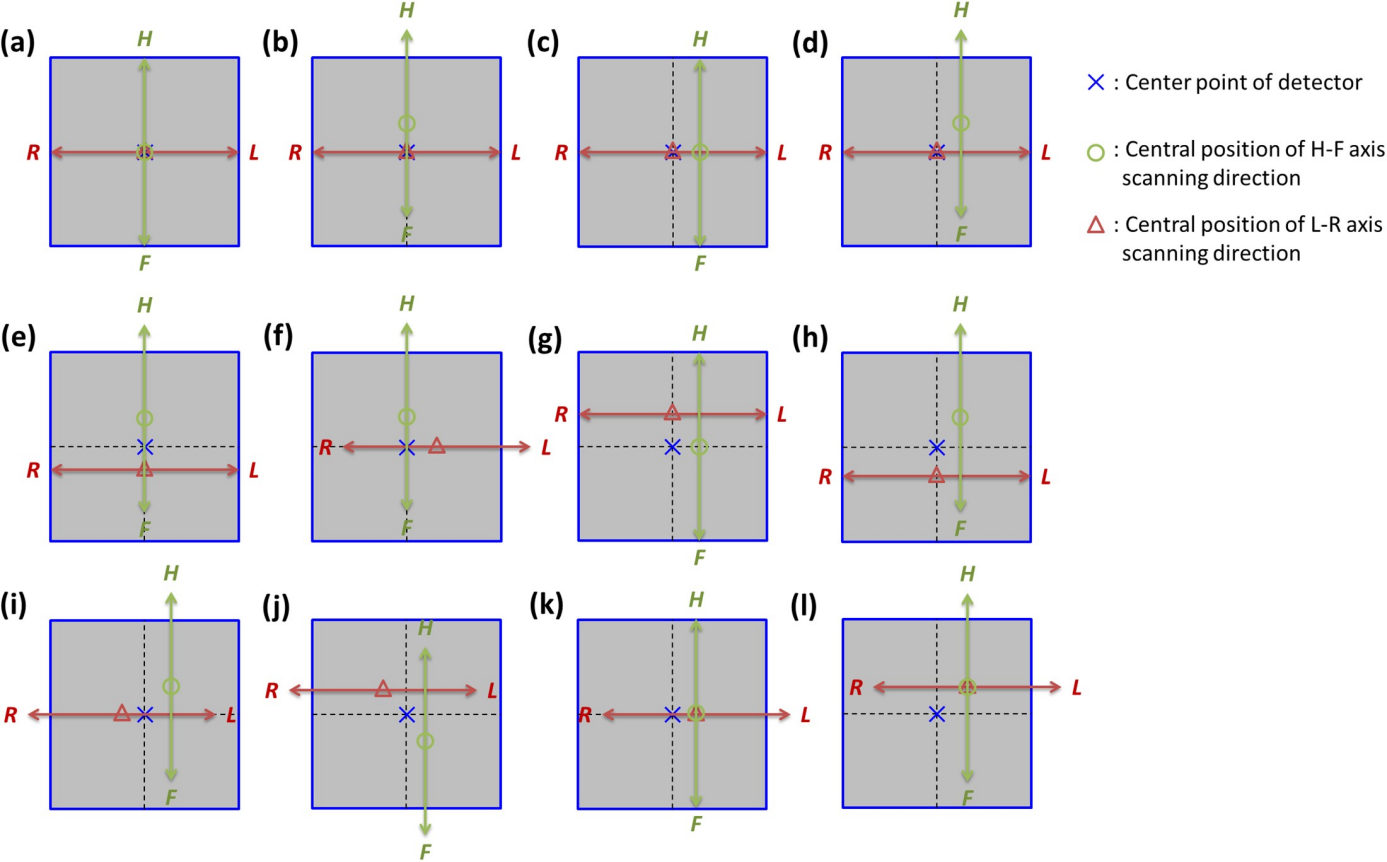
Various misalignment scenarios of axis shift for the tomosynthesis system with dual-axis scanning geometry. (a) the ideal situation. (b) HF-axis shift in the HF-axis scanning direction. (c) LR-axis shift in the HF-axis scanning direction. (d) both HF-axis and LR-axis shifts in the HF-axis scanning direction. (e) HF-axis shift in the HF-axis and LR-axis scanning directions, respectively. (f) the HF axis exhibits HF-axis shift, and the LR axis exhibits LR-axis shift. (g) the HF axis exhibits LR-axis shift, and the LR axis exhibits HF-axis shift. (h) only the LR-axis position of LR-axis scanning is appropriate for the system geometry. (i) only the HF-axis position of LR-axis scanning is appropriate for the system geometry. (j) none of the axis positions are suitable for the system geometry. (k) and (i) are special cases in which the center points of the two scanning axes are the same but different from the center of the detector.

Fig 3 shows the different scenarios of misalignments of axis shift for dual-axis scanning geometry without axis tilt. Fig 3(a) presents the ideal situation, in which the center points of the two scanning axes and the center of the detector are at the same point. The scenarios in which the system has only a single-axis shift are presented in Fig 3(b) and 3(c). The scenarios in which the system has a shift in two axes are depicted in Fig 3(d) to 3(g). The scenarios in which the system has a shift in three axes are depicted in Fig 3(h) and 3(i), and the scenario in which the system has a shift in four axes is presented in Fig 3(j). Fig 3(k) and 3(l) are special cases in which the center points of the two scanning axes are the same but different from the center of the detector.

**Fig 4:**
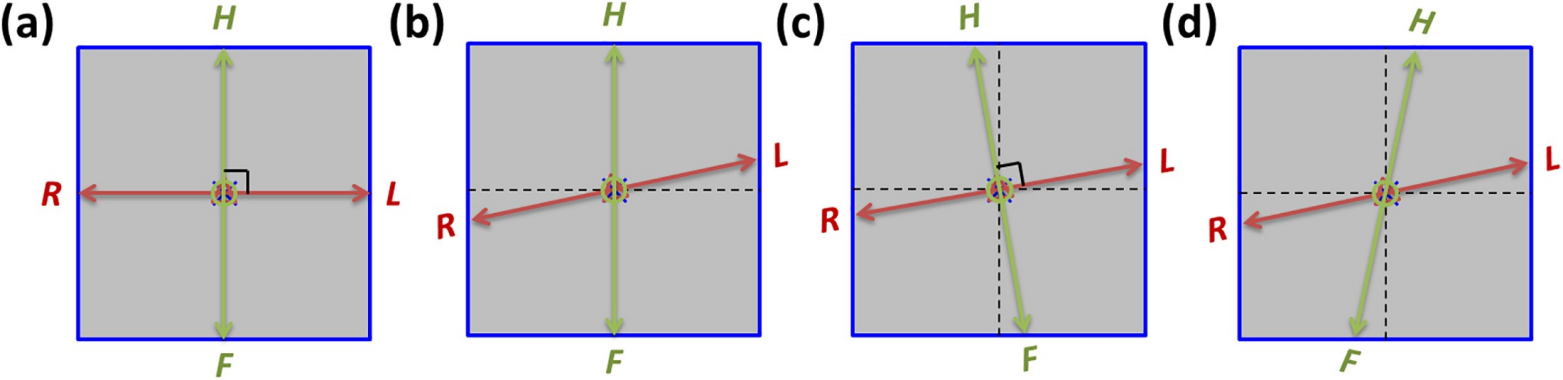
Various misalignment scenarios of axis tilt for the tomosynthesis system with dual-axis scanning geometry. (a) the ideal situation. (b) the system with single-axis tilt for either HF-axis tilt or LR-axis tilt. (c) the rotation of the scanning coordinate system, in which the HF and LR axes are still in two perpendicular directions. (d) the system with dual-axis tilt, which means that none of the axes are parallel to the reference axes and that the two axes are not perpendicular to each other.

Fig 4 presents the different scenarios of misalignments of axis tilt for dual-axis scanning geometry without axis shift. The center points of the two scanning axes and the center of detector are at the same point. Fig 4(a) presents the ideal situation, in which HF and LR axes are the same as the reference axes (dash lines) of the coordinate system. Fig 4(b) depicts the single-axis tilt of the system. Fig 4(c) displays the rotation of the scanning coordinate system. Fig 4(d) presents the system with dual-axis tilt.

**Fig 5:**
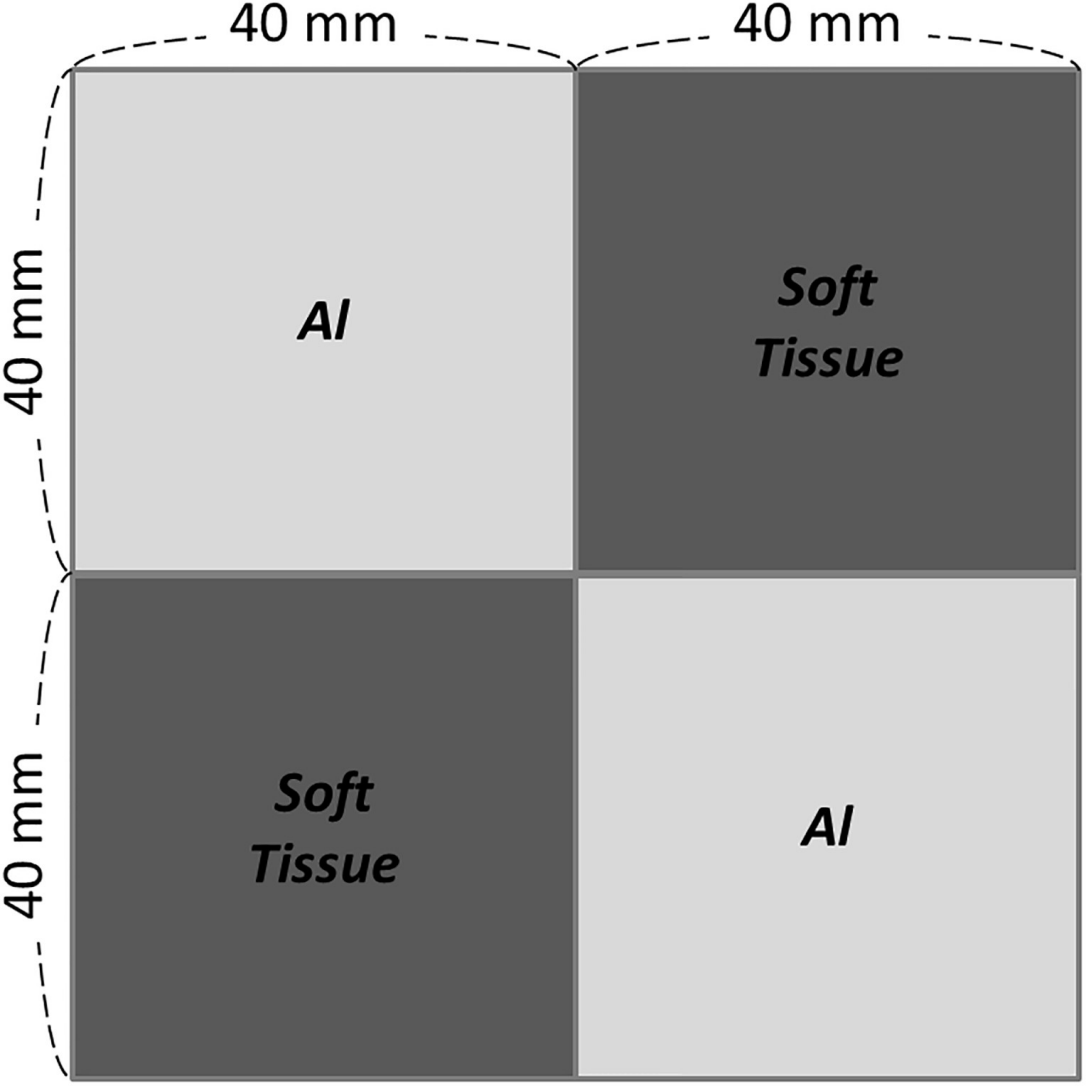
Schematic of simple digital phantom to evaluate the level of influence of system geometric misalignments.

To evaluate the effects of geometric misalignments on different scenarios of tomosynthesis reconstruction images, we simulated X-ray projection images with the same geometry of TomoDR. The digital phantom with simple geometry is depicted in Fig 5. The phantom consists of two materials with various characteristics, one being the soft tissue (ρ = 1.03 g/cm³) and the other being aluminum (ρ = 2.699 g/cm³), which can be used to evaluate the level of influence of system geometric misalignments. The size of each block is 40 × 40 mm^2^, and the thickness is 50 mm. For the simulation geometry setup, the distance from the bottom of the phantom to the surface of the image receptor was 150 mm, which was set to imitate the position of the mass inside the chest.

### Projection matrix-based geometric calibration method with iterative optimization

**Fig 6:**
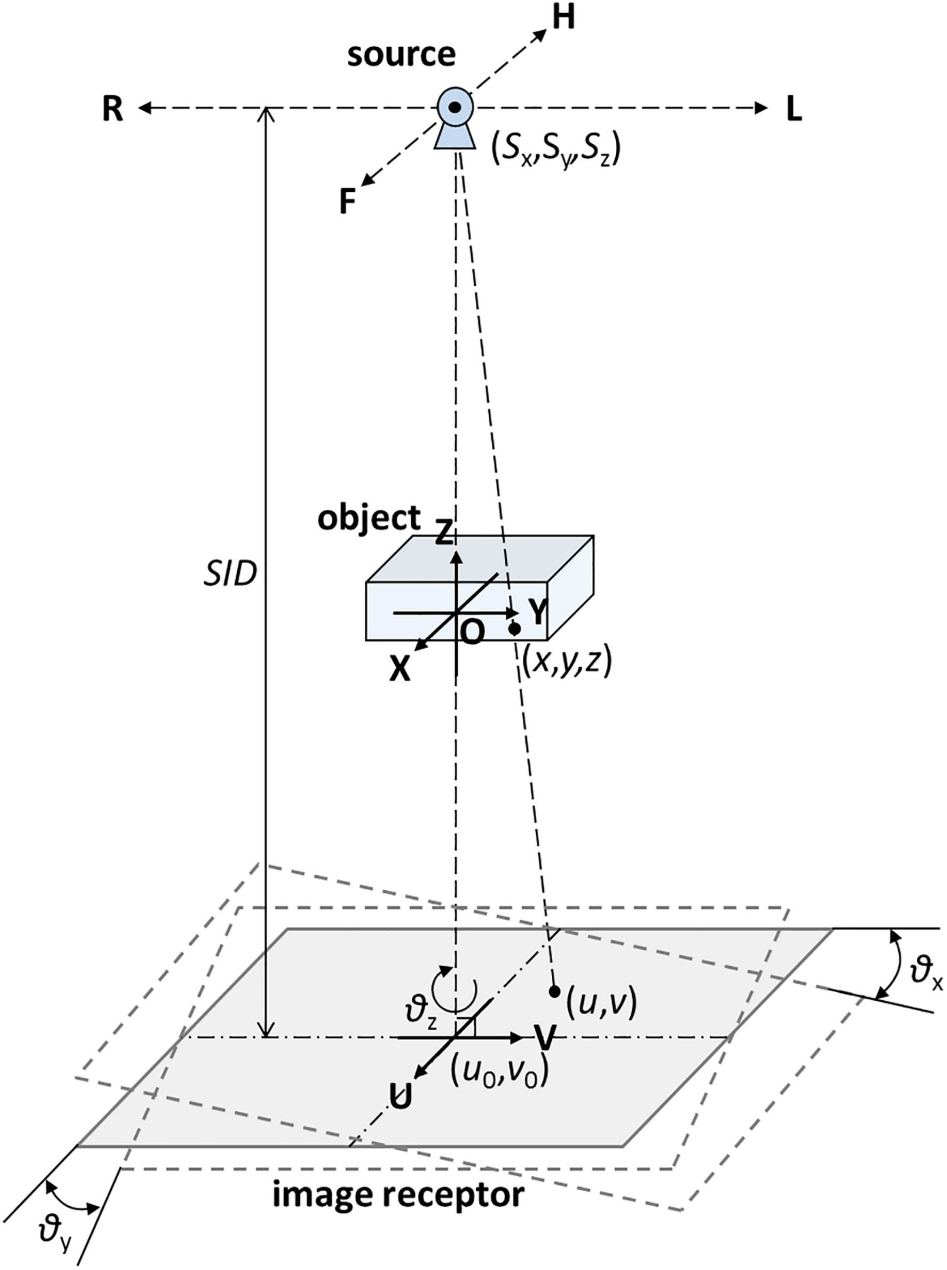
Schematic of TomoDR imaging geometry. Including the source to image receptor distance (*SID*), the central ray offsets (*u*_*0*_, *v*_*0*_), the source position (*S*_*x*_, *S*_*y*_, *S*_*z*_), and the rotation angles (*θ*_*x*_, *θ*_*y*_, *θ*_*z*_) of the image receptor.

For the geometric coordinate system of TomoDR, the source (*S*_*x*_, *S*_*y*_, *S*_*z*_) has two sweep directions: the HF axis and LR axis. During the imaging process, the coordinate (*x*, *y*, *z*) in the object space is projected to the coordinate (*u*, *v*) in the detector plane. The schematic of the system geometric relationship is displayed in Fig 6. For homogeneous coordinate systems, a general equation for mapping the coordinates of the 3D object space to the 2D detector plane can be expressed in the projection matrix-based form as follows. For more details, see [10, 15].

$$w{\left[u,v,1\right]}^{T}=\text{P}{\left[x,y,z,1\right]}^{T}$$ (1)

where **P** is a 3 × 4 projection matrix that relates the mapping of the object coordinate (*x*, *y*, *z*) to its projection coordinate (*u*, *v*) in the detector plane. *w* is a distance weighting factor. The projection matrix **P** can be factorized as follows:

P=K[R|t] (2)

where **K** is a 3 × 3 intrinsic matrix, **R** is a 3 × 3 rotation matrix, and **t** is a 3 × 1 translation vector.

$$K=\left[\begin{array}{ccc} \frac{SID}{p_{u}} & 0 & u_{0}\\ 0 & \frac{SID}{p_{v}} & v_{0}\\ 0 & 0 & 1 \end{array}\right]$$ (3)

where *SID* is the distance from the X-ray source to the image receptor (unit: mm), *p*_*u*_ and *p*_*v*_ are the pixel height and width of the image receptor (unit: mm), respectively, and *u*_0_ and *v*_0_ are the coordinates of the intersection point in relation to the center of the X-ray and image receptor, respectively. (unit: mm)

$$R=\left[\begin{array}{ccc} cos\theta_{z}cos\theta_{y} & -sin\theta_{z}cos\theta_{x}+cos\theta_{z}sin\theta_{y}sin\theta_{x} & sin\theta_{z}sin\theta_{x}+cos\theta_{z}sin\theta_{y}cos\theta_{x}\\ sin\theta_{z}cos\theta_{y} & cos\theta_{z}cos\theta_{x}+sin\theta_{z}sin\theta_{y}sin\theta_{x} & -{cos\theta }_{z}sin\theta_{x}+sin\theta_{z}sin\theta_{y}cos\theta_{x}\\ -sin\theta_{y} & cos\theta_{y}sin\theta_{x} & cos\theta_{y}cos\theta_{x} \end{array}\right]$$ (4)

where *θ*_*x*_, *θ*_*y*,_ and *θ*_*z*_ are the Euler angles indicating the orientation of the image receptor along the x, y, and z axes in the object coordinate system, respectively (unit: degrees). The translation vector **t** consists of the following three elements:

$$t={\left[t_{x}{,t}_{y},t_{z}\right]}^{T}$$ (5)

Where *t*_*x*_, *t*_*y*_, and *t*_*z*_ denote the distance in shift between the object and source coordinate system.

To further improve the calculation accuracy of the projection matrix **P**, we used a nonlinear least-squares method to iteratively minimize the square distance between the measured marker coordinates (*u*_*i*_, *v*_*i*_) and their reprojected coordinates (*u*_*i*_(**P**), *v*_*i*_(**P**)). The reprojected coordinates of the markers in the calibration phantom can be calculated by Eq (1). The projection matrix **P** is adjusted to minimize the square distance between (*u*_*i*_ − *u*_*i*_(**P**)) and (*u*_*i*_ − *u*_*i*_(**P**)) to obtain the optimized **P**. The algorithm we used was the Levenberg–Marquardt algorithm [28], and the objective function was as follows:

$$E=\sum_{i=1}^{N}\left[{\left(u_{i}-u_{i}(P)\right)}^{2}+{\left(v_{i}-v_{i}(P)\right)}^{2}\right]$$ (6)

where *u*_*i*_ and *v*_*i*_ are measured marker coordinates, *u*_*i*_(**P**) and *v*_*i*_(**P**) are reprojected marker coordinates using the projection matrix approach, and *N* is the number of markers in the object. The initial guess of the projection matrix **P** was calculated by using the direct linear transformation (DLT) algorithm in Eq (1).

With the known matrices **P**, **K**, and **R**, the geometric parameters can be extracted and *u*_0_ and *v*_0_ can be expressed as follows:

$$u_{0}=K_{13}$$ (7)

$$v_{0}=K_{23}$$ (8)

where *u*_0_ and *v*_0_ are the central ray offsets, and *K*_13_ and *K*_23_ are the elements of the intrinsic matrix **K**.

The parameter *SID* is as follows:

$$SID=K_{11}p_{u}=K_{22}p_{v}$$ (9)

The rotation angles of the image receptor are as follows:

$$\theta_{x}=arctan(R_{32},R_{33})$$ (10)

$$\theta_{y}=arcsin(-R_{31})$$ (11)

$$\theta_{z}=arctan(R_{21},R_{11})$$ (12)

The source position (*S*_*x*_, *S*_*y*_, *S*_*z*_) can be calculated using the following constraint:

PS=0 (13)

This equation can be solved using the singular value decomposition method to acquire the vector **S**:

$$S={\left[S_{x}{,S}_{y},S_{z},1\right]}^{T}$$ (14)

where *S*_*x*_, *S*_*y*_, and *S*_*z*_ are the coordinates of the X-ray source.

### The simulation setup for X-ray imaging

To evaluate the accuracy of the geometric calibration algorithm, we developed a program to simulate 2D X-ray projection images from 3D objects. The X-ray imaging simulation we used is numerical method, and the code is written in C++ programs. We used the ray-tracing algorithm which is based on a simple method for calculating line-integrals. [29] We only simulated the interaction of the photon and the object without photon scattering. The input parameters of this program consist of the X-ray energy, the object material and geometry, the image receptor properties, and the system geometry and scanning trajectory. The outputs are the 2D X-ray projection images at various source positions. The parameters of simulation are set in the simple condition because the projections are only used to evaluate the geometric parameters. The focal spot of the X-ray tube is an ideal point source, and the X-ray with 90keV monolithic energy covers the entire image receptor during the imaging process. The image receptor has perfect detection efficiency, the modulation transfer function (MTF), and the noise power spectrum (NPS). The system geometry and scanning trajectory are the same as the TomoDR setup. The digital phantom design is described in the following section.

### Design of the calibration phantom and data analysis

**Fig 7:**
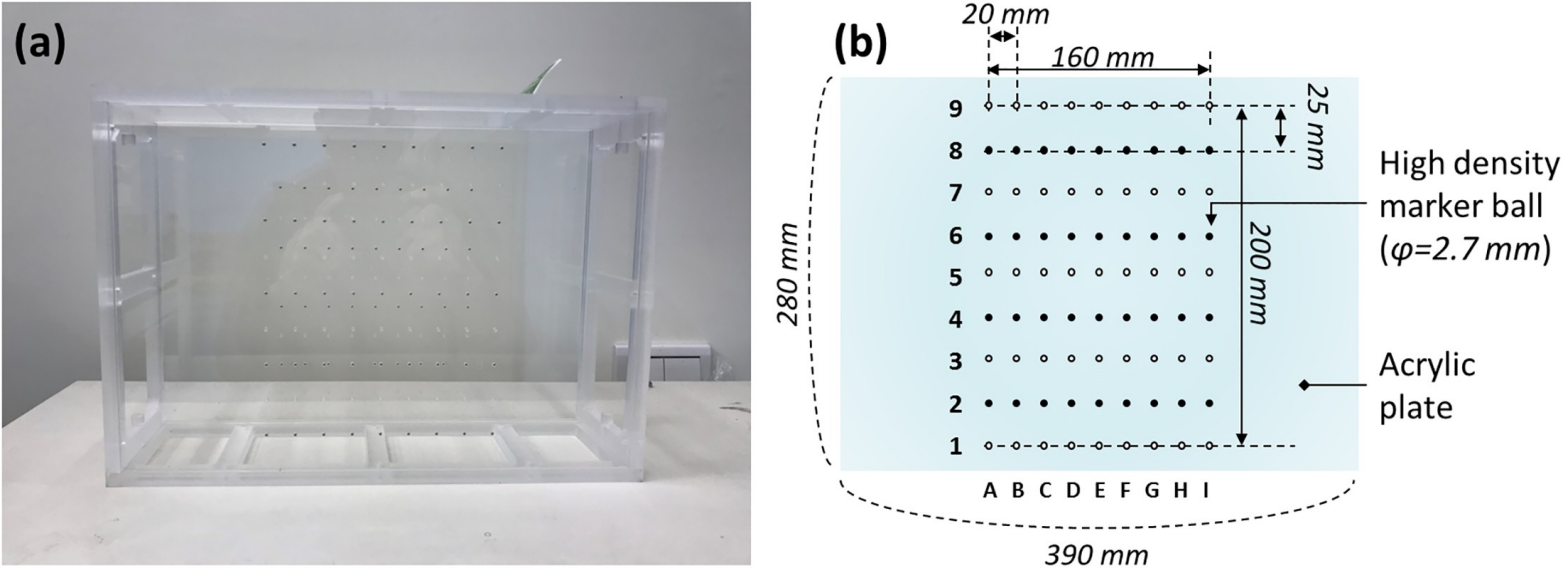
Geometric calibration phantom. (a) Front view of the geometric calibration phantom. (b) Schematic of markers placement in the phantom.

To calculate the aforementioned geometric parameters, we designed a calibration phantom containing 81 stainless steel spheres of 2.7 mm diameter and two acrylic plates (ρ = 1.18 g/cm³), as displayed in Fig 7. The size of beads was optimized by considering the system magnification factor and the pixel size of the image receptor. In general, a large volumetric coverage of markers is preferable for mitigating the calculation error for the phantom-based geometric calibration method [19, 25]. Hence, we designed a suitable beads distribution to fit the geometry of TomoDR. Fig 7(a) indicates that the bead markers are arranged in two parallel plates with 45 (non-solid spheres) and 36 (solid spheres) beads in the top and bottom planes, respectively. The distance between bead markers in XY directions are shown in Fig 7(b), and the distance between bead markers in Z direction is 100 mm. The calibration procedure was sensitive to errors in the position information; therefore, the exact coordinates of each bead should be known for the subsequent extraction of the geometric parameters. To extract the geometric parameters accurately, the centroid positions were used as the projected bead coordinates on the projection images [30]. The geometric calibration program code written in MATLAB was used to calculate the projected bead coordinates, projection matrix, and geometric parameters.

**Fig 8:**
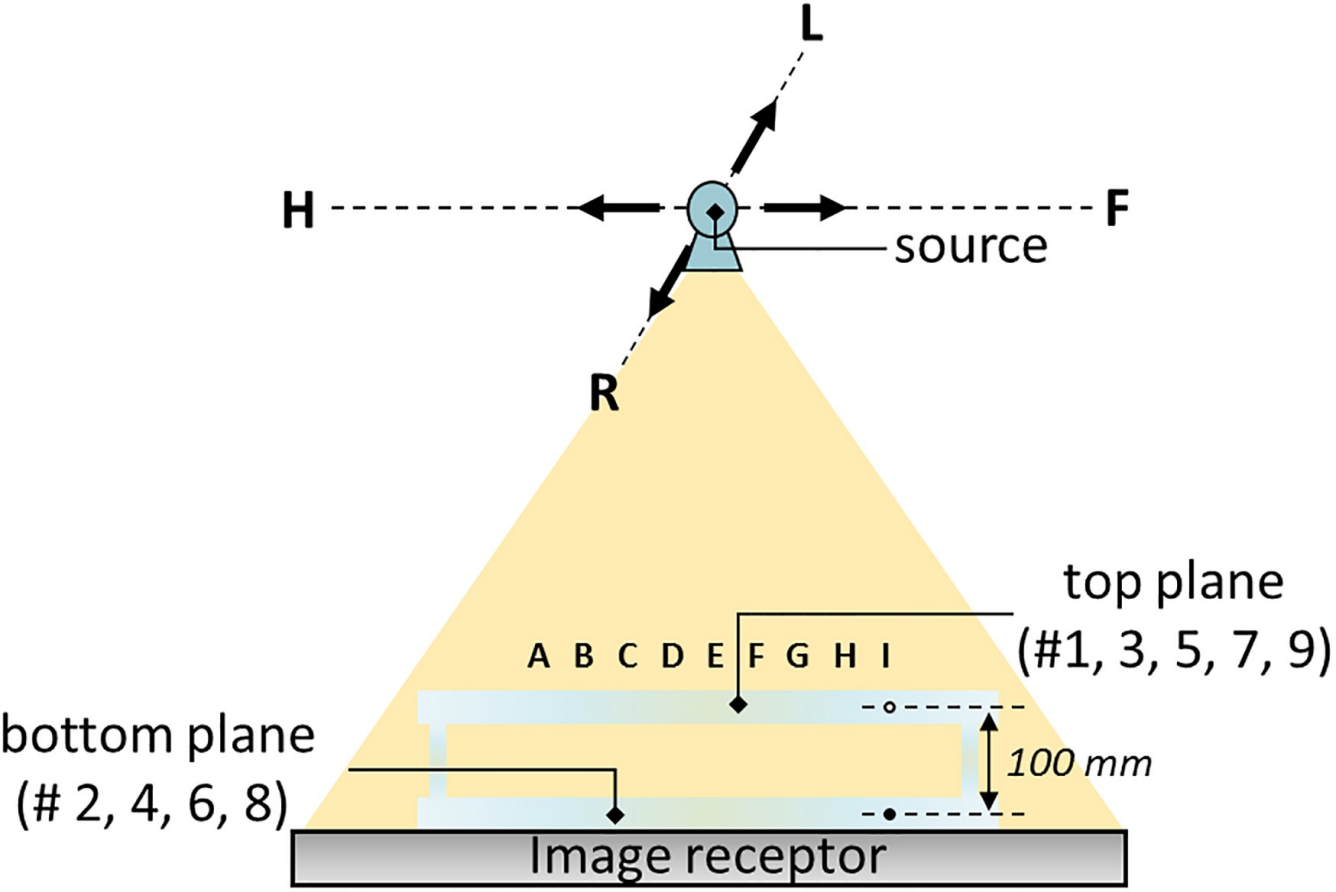
The setup of geometric calibration experiment. The odd numbers of rows are placed in top plane, which are denoted as non-solid spheres. And the even numbers of rows are placed in bottom plane, which are denoted as solid spheres. The vertical distance between non-solid and the solid spheres is 100 mm.

**Fig 9:**
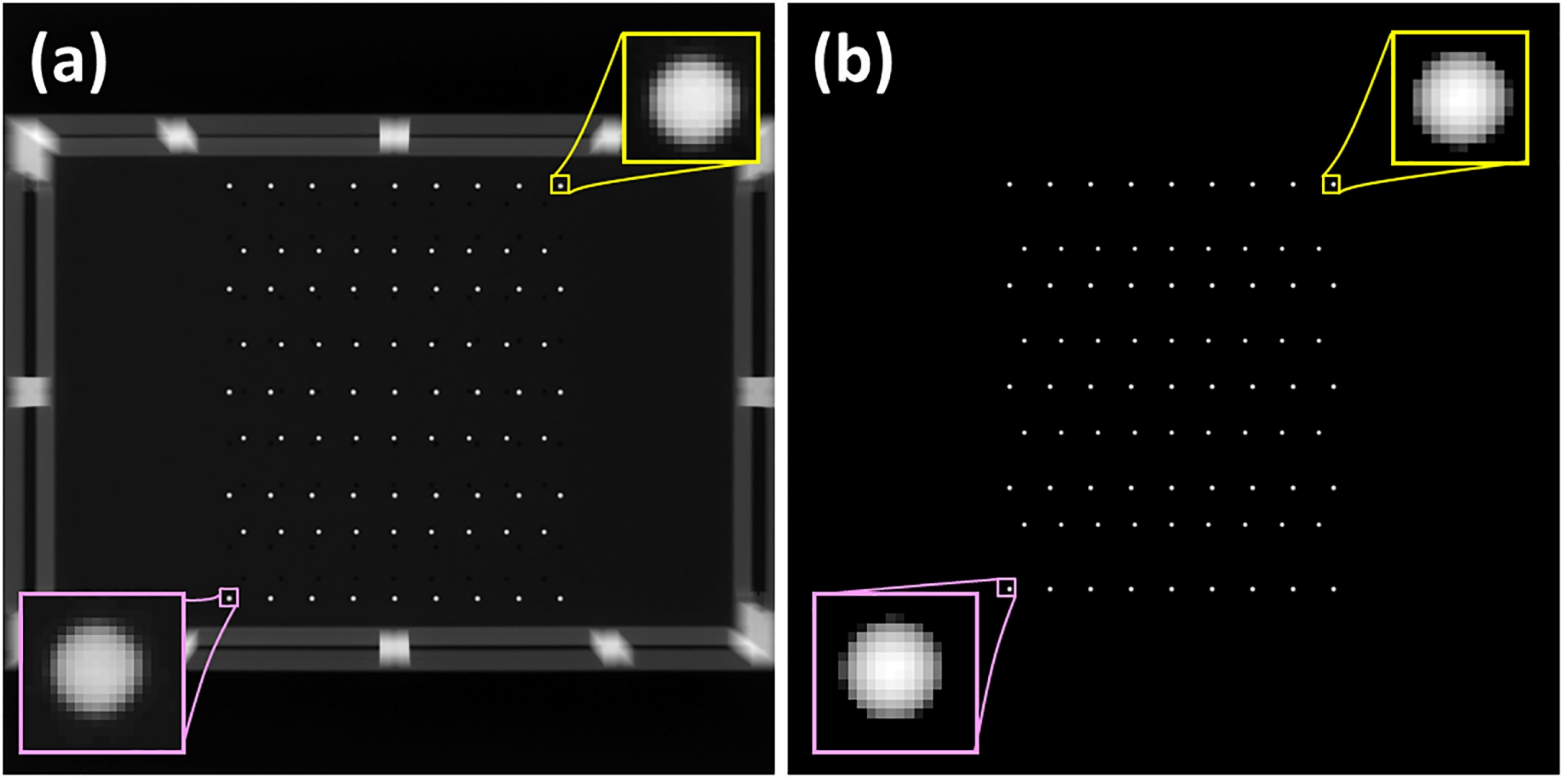
The experimental and simulated projection images of the calibration phantom. The position of the source (*S*_*x*_, *S*_*y*_, *S*_*z*_) is (0, 0, 1100 mm). The central ray of source pass through the center of the image receptor, and it is perpendicular to the image receptor. (a) Experimental result. (b) Simulated result.

The geometric calibration procedure was implemented in the following steps. First, the calibration phantom was placed on the image receptor or table surface to acquire projection images displayed in Fig 8. To imitate a realistic imaging situation, we used the chest scanning protocol with 61 and 31 projections along the HF-axis and LR-axis directions, respectively. The phantom projection image of the experiment and simulation are presented in Fig 9(a) and 9(b), respectively. The geometry of the phantom and imaging system were the same for the experiment and simulation. Second, the bead centroids of the projection images were calculated to be the coordinates of 2D projection markers. Third, the projection matrix was iteratively calculated by mapping the relationship between the known 3D coordinates of the markers and the 2D projection coordinates of these markers on the image receptor. Finally, the geometric parameters were extracted using projection matrix decomposition.

### Experimental assessment for the geometric calibration performance

To evaluate the performance of the geometric calibration method, we used an anthropomorphic chest phantom (Model: LUNGMAN, Kyoto Kagaku, Japan) and the line pair phantom (Model: Type-52, Hüttner Röntgenteste, Germany) to evaluate the image quality and the MTF of the reconstructed images, respectively. The method of the MTF calculation we used is the slit technique with bar-pattern images [31–32] The scanning protocol of the line pair phantom is the same as the chest tomosynthesis imaging (100 kV and 0.16 mAs for each projection), and we reconstructed 92 projection images in a 1024 × 1024 × 100 pixels matrix with a voxel size of 0.278 × 0.278 × 1 mm³. The distance from the first slice to the detector surface is 70 mm, and we chose the slice number 16 to calculate the MTF with and without calibration.

## Results

### The simulation results of the extracted geometric parameters of tomosynthesis with dual-axis scanning geometry

To develop a realistic imaging situation, the simulated system geometry was the same as TomoDR. A coordinate system was defined as illustrated in Fig 6. During an imaging process, the object and image receptor are stationary, and only the X-ray source moves along the HF and LR axes in step and shoot imaging mode.

For quantitative analysis of the geometric calibration method, the projection images of the calibration phantom were simulated using the aforementioned system geometry. We used the known 3D coordinates of the beads and their 2D coordinates on the projection images to calculate the geometric parameters. The geometric parameters that were input into the simulation were compared with the extracted values to validate their accuracy. The input and extracted parameters for both the HF and LR axes are listed in Table 1.

**Table 1:** Comparisons of the input geometric parameters and extracted parameters in the simulation study.

			*SID* [mm]	*u*_0_ [mm]	*v*_0_ [mm]	*S*_*x*_ [mm]	*S*_*y*_ [mm]	*S*_*z*_ [mm]	*θ*_*x*_ [deg.]	*θ*_*y*_ [deg.]	*θ*_*z*_ [deg.]
**HF**	**Input parameters**		1120	Variable	0	Variable	0	1100	0	0	0
	**Extracted parameters**	**Mean**	1120.19	N/A	1.48E-11	N/A	-3.47E-12	1100.18	-9.66E-13	3.26E-03	-1.05E-14
		**STD**	0.07		6.21E-12		2.60E-12	0.06	3.22E-13	2.32E-03	4.13E-14
		**MAD**	0.19	0.069	1.48E-11	0.028	3.86E-12	0.18	9.66E-13	3.26E-03	3.65E-14
**LR**	**Input parameters**		1120	0	Variable	0	Variable	1100	0	0	0
	**Extracted parameters**	**Mean**	1120.03	2.12E-11	N/A	1.54E-12	N/A	1100.02	-4.84E-13	9.96E-13	9.43E-15
		**STD**	0.16	9.33E-12		9.91E-13		0.14	5.53E-03	4.66E-13	7.35E-14
		**MAD**	0.13	2.12E-11	0.091	1.73E-12	0.013	0.12	4.48E-03	9.96E-13	5.92E-14

During the system-scanning process, the object, *SID*, height position of the X-ray source, and image receptor were stationary. The values for central ray offset (*u*_0_) and source position (*S*_*x*_) were variable because the U-axis of the image receptor and X-axis of the system coordinates were parallel to the HF axis (Fig 6); moreover, *v*_0_ and *S*_*y*_ were also variable in the LR-axis scanning, as indicated in Fig 6. We therefore used the index mean absolute deviation (MAD) in the statistic field to describe the calculation error of extracted parameters, which were computed as the absolute mean error of input geometric parameters and extracted parameters. Table 1 reveals that the values for the extracted geometric parameters were similar to the input values, and the maximum MAD of the calculated image receptor orientation (*θ*_*x*_, *θ*_*y*_, *θ*_*z*_) was less than 0.01°. The majority of the MAD values for the extracted parameters were less than one pixel size of the image receptor (0.139 mm), with the exception of the *SID* and *S*_*z*_. However, the maximum MAD of these two parameters was less than 0.2 mm, which was smaller than their input values. Therefore, In the simulation study, the results of the extracted geometric parameters used in the geometric calibration method were extremely close to the true values of the system geometry (the relative error of the input and extracted values was 0.02%).

### The simulation results of the dual-axis scanning geometry scanner with geometric misalignments

**Fig 10:**
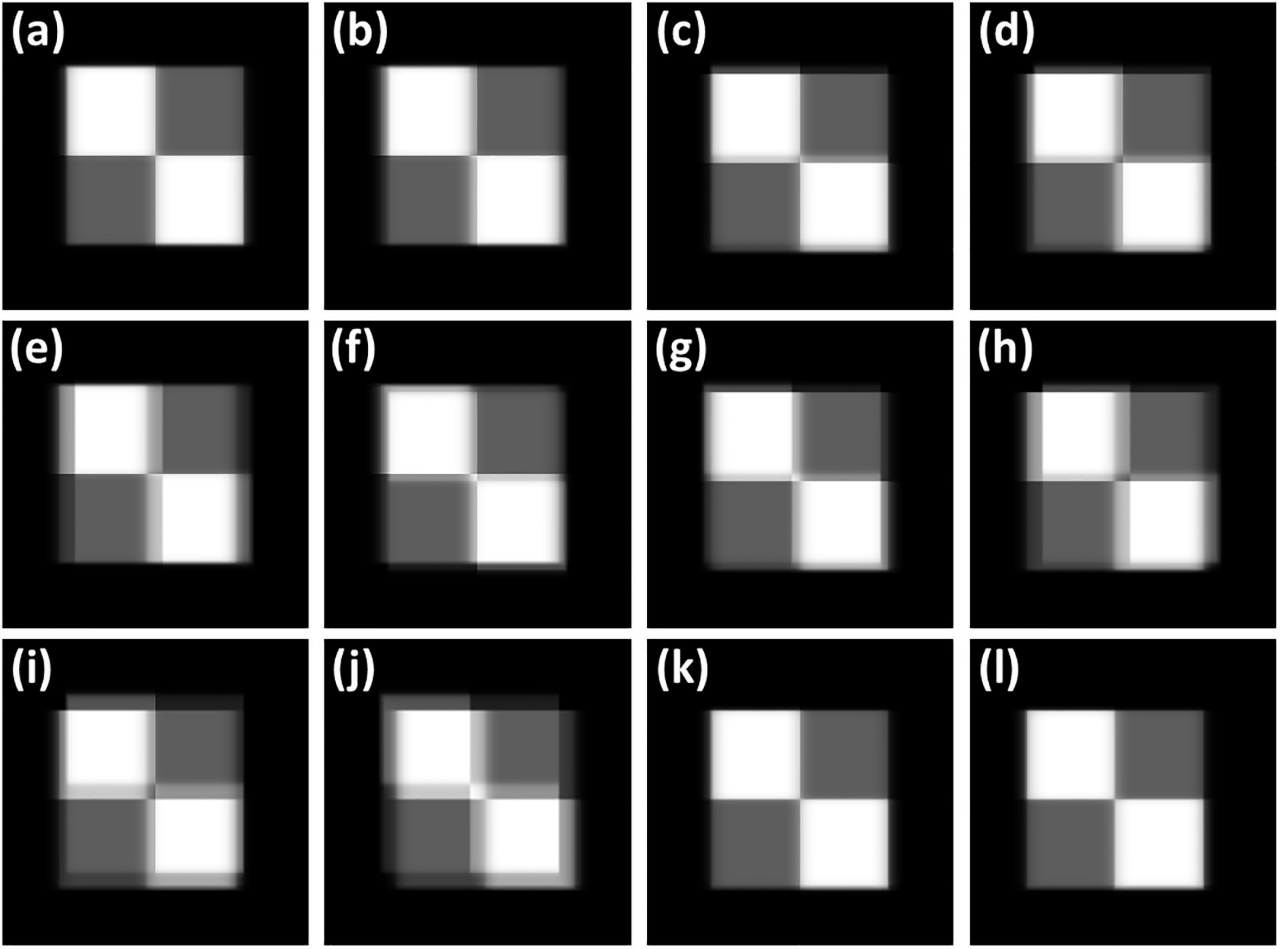
Simulated tomosynthesis reconstruction images with the various misalignment scenarios of axis shift displayed in Fig 3.

**Fig 11:**
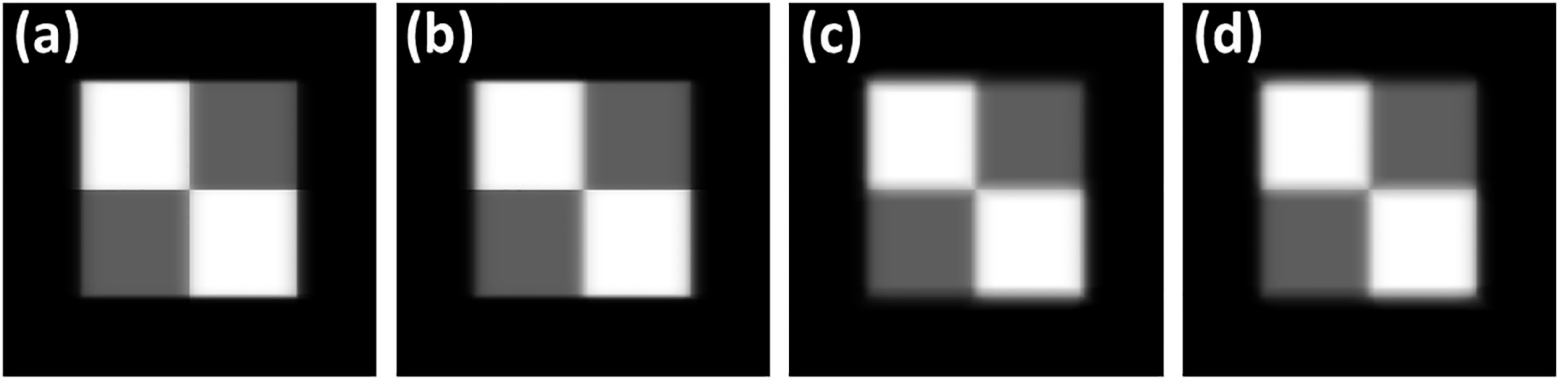
Simulated tomosynthesis reconstruction images with the various misalignment scenarios of axis tilt presented in Fig 4.

The reconstructed images of various misalignment scenarios shown in Figs 3 and 4 are presented in Figs 10 and 11, respectively, to evaluate the influences of these misalignment scenarios. The dimensions of the reconstructed images were 1024 × 1024 × 10, the voxel size was 0.5 × 0.5 × 5 mm³, the axis shift was 20 mm for each axis for all cases, and the central slice was selected for evaluation of the image quality. Fig 10(a) is the reconstructed image for the ideal situation, which can be regarded as the reference image. Figs 10(b) to 10(j) present the reconstructed images with artifacts in varying degrees. The poor image quality of the reconstructed images can be observed in Fig 10(j), which reveals that the system demonstrated shift in four axes. The misalignment scenario displayed in Fig 10(j) was used to evaluate the performance of the geometric calibration method with axis shift in the following content. Fig 10(k) and 10(l) depict that adequate image quality can be observed through mismatching of the two scanning axes and reference axes. Moreover, this result revealed that the influence of the axis shift on image quality for the single-axis scanning geometry was almost negligible.

Fig 11(a) also displays the reconstructed image for the ideal situation presented in Fig 10(a). Fig 11(b) to 11(d) display the reconstructed images with a 5° tilt of each axis, as exhibited in Fig 4(b) to 4(d). The reconstructed images with the poor image quality are presented in Fig 11(c) and 11(d), which reveal that the system demonstrated tilt on two axes. The misalignment scenario displayed in Fig 11(d) was also used to evaluate the performance of the geometric calibration method with axis tilt in the following content.

**Fig 12:**
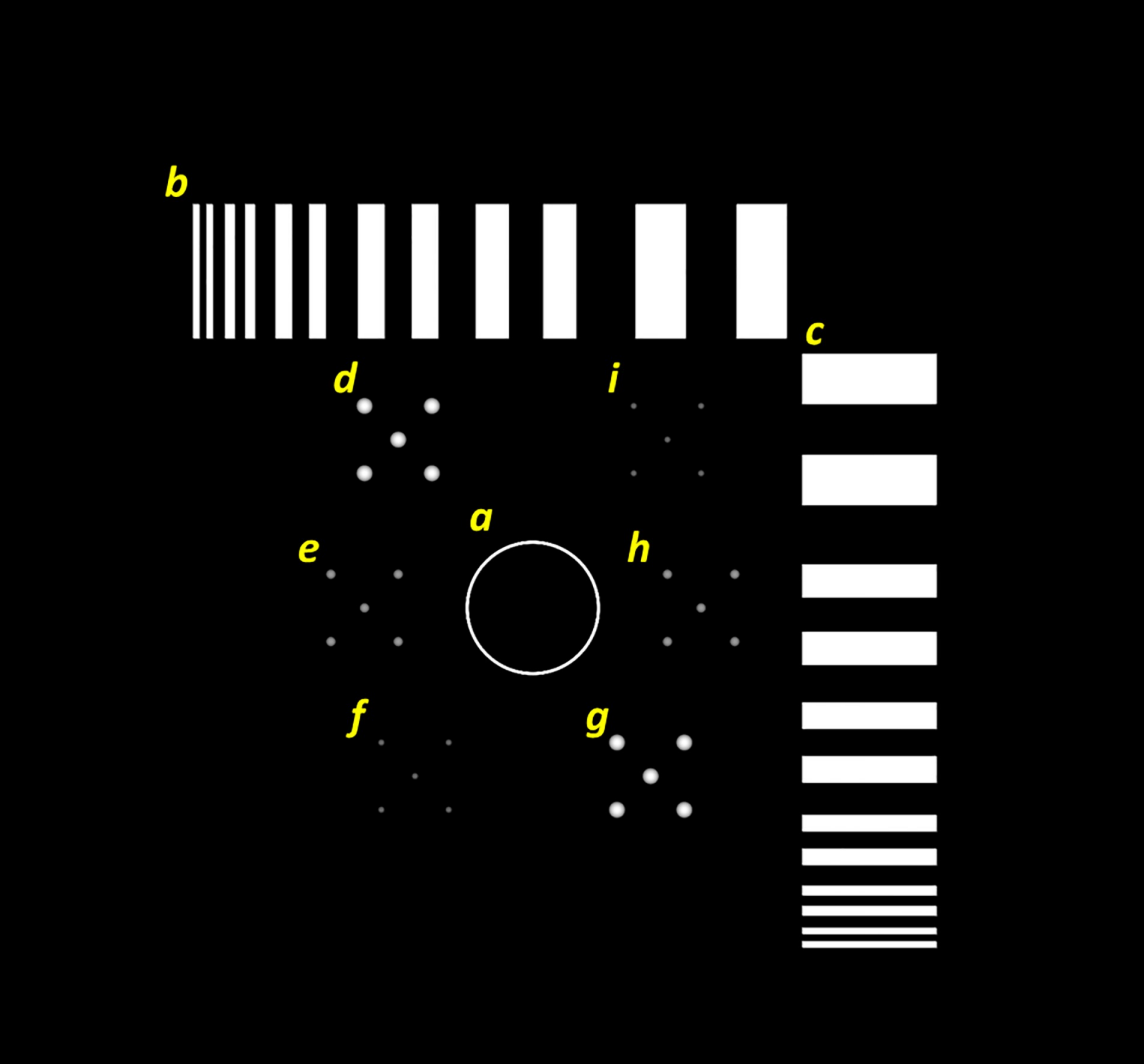
Simulated projection image of the digital phantom for image quality assessment.

To further assess the image quality of the reconstructed images with system geometric misalignments, we designed a digital phantom with different test objects, as demonstrated in Fig 12. The phantom consisted of three major components: one circular object (#a), two sets of linearly paired objects (#b, #c), and six groups of pecks objects (#d to #g). The thickness of the circular object was 5 mm, and the inner and outer radius was 38 and 40 mm, respectively. The spatial frequencies of the linear pairs were 0.03, 0.05, 0.06, 0.10, 0.17, and 0.25 lp/mm, with a thickness of 5 mm. The diameter of the peck objects were 2, 3, and 5 mm. All the objects were made of lead.

To simulate the geometric misalignments of the imaging equipment for axis shift scenarios, the most extreme situation was selected to evaluate the influence on the reconstructed images (Fig 3(j)). The two axes of the X-ray source were offset by 0, 1, 3, 5, or 10 mm, and the calibration process of TomoDR was as follows. First, the digital image quality assessment phantom was used to acquire various angle projection images with a chest imaging protocol, and five sets of projection images were obtained according to the aforementioned axis shift value. Second, the calibration phantom was utilized to extract geometric parameters for the system, and five sets of geometric parameters were obtained. Finally, projection images were reconstructed based on the SART algorithm with and without calibrated geometric parameters to evaluate the efficiency of the geometric calibration algorithm.

**Fig 13:**
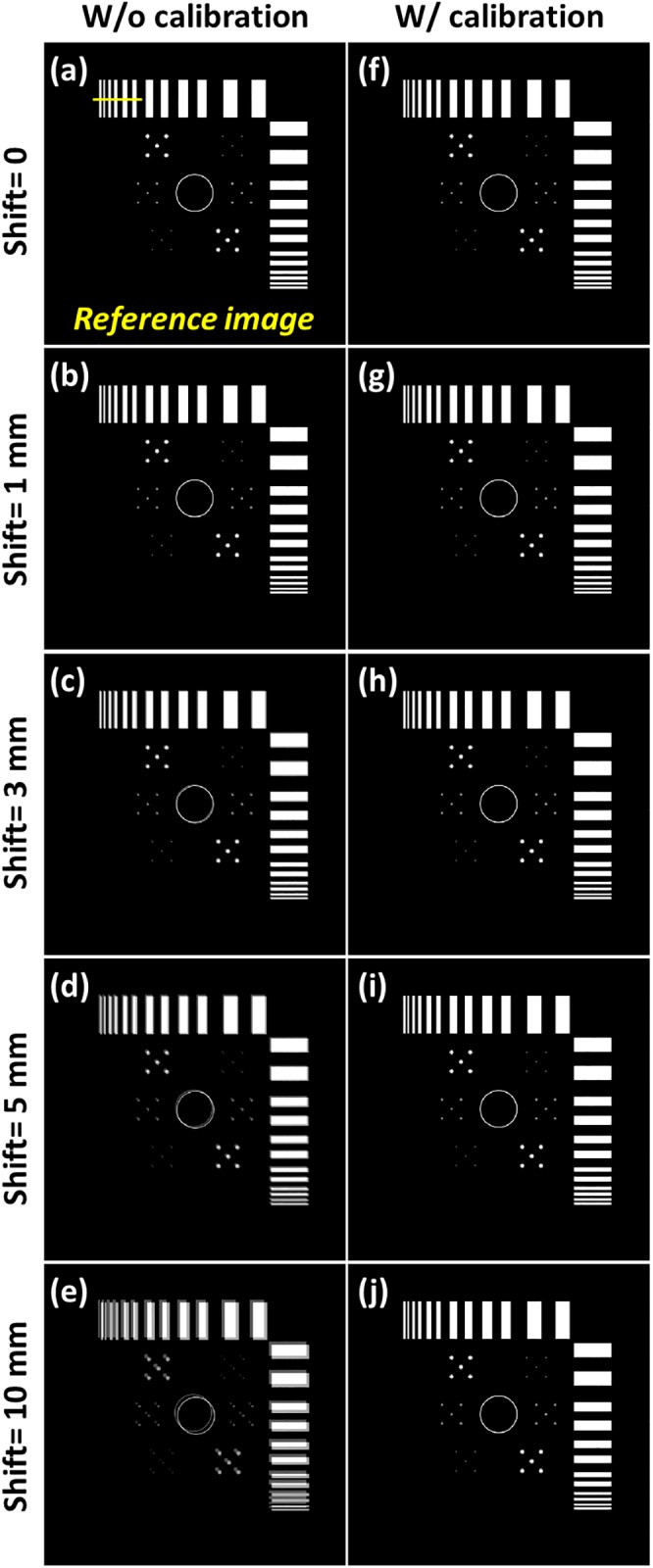
Simulated tomosynthesis reconstruction images of various values of axis shift with and without geometric calibration. (a) Reconstructed image with the ideal situation and no geometric calibration. (b) axis shift = 1 mm; (c) axis shift = 3 mm; (d) axis shift = 5 mm; (e) axis shift = 10 mm. (f) Reconstructed image with the ideal situation and geometric calibration. (g) Reconstructed image of (b) after calibration. (h) Reconstructed image of (c) after calibration. (i) Reconstructed image of (d) after calibration. (j) Reconstructed image of (e) after calibration.

**Fig 14:**
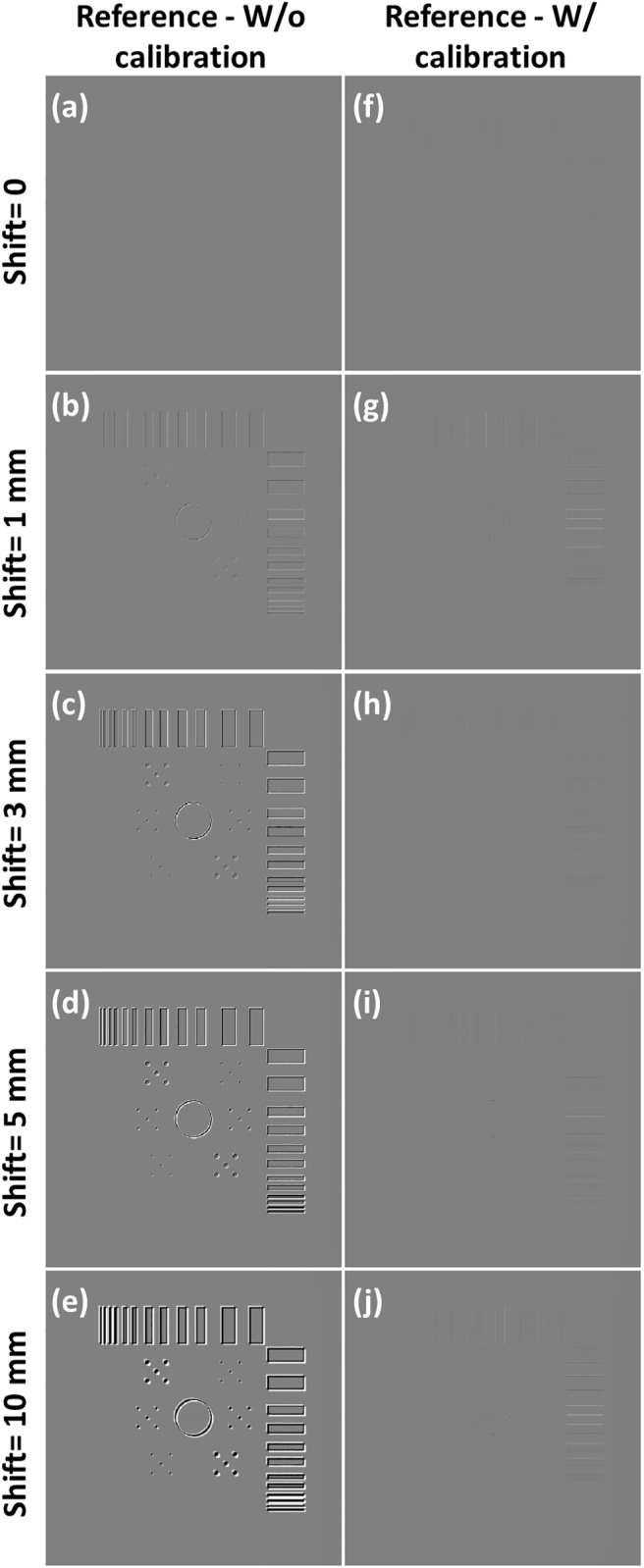
Difference images of the reference image and simulated reconstruction images of axis shift presented in Fig 13. Images are displayed between −0.2 and +0.2.

**Fig 15:**
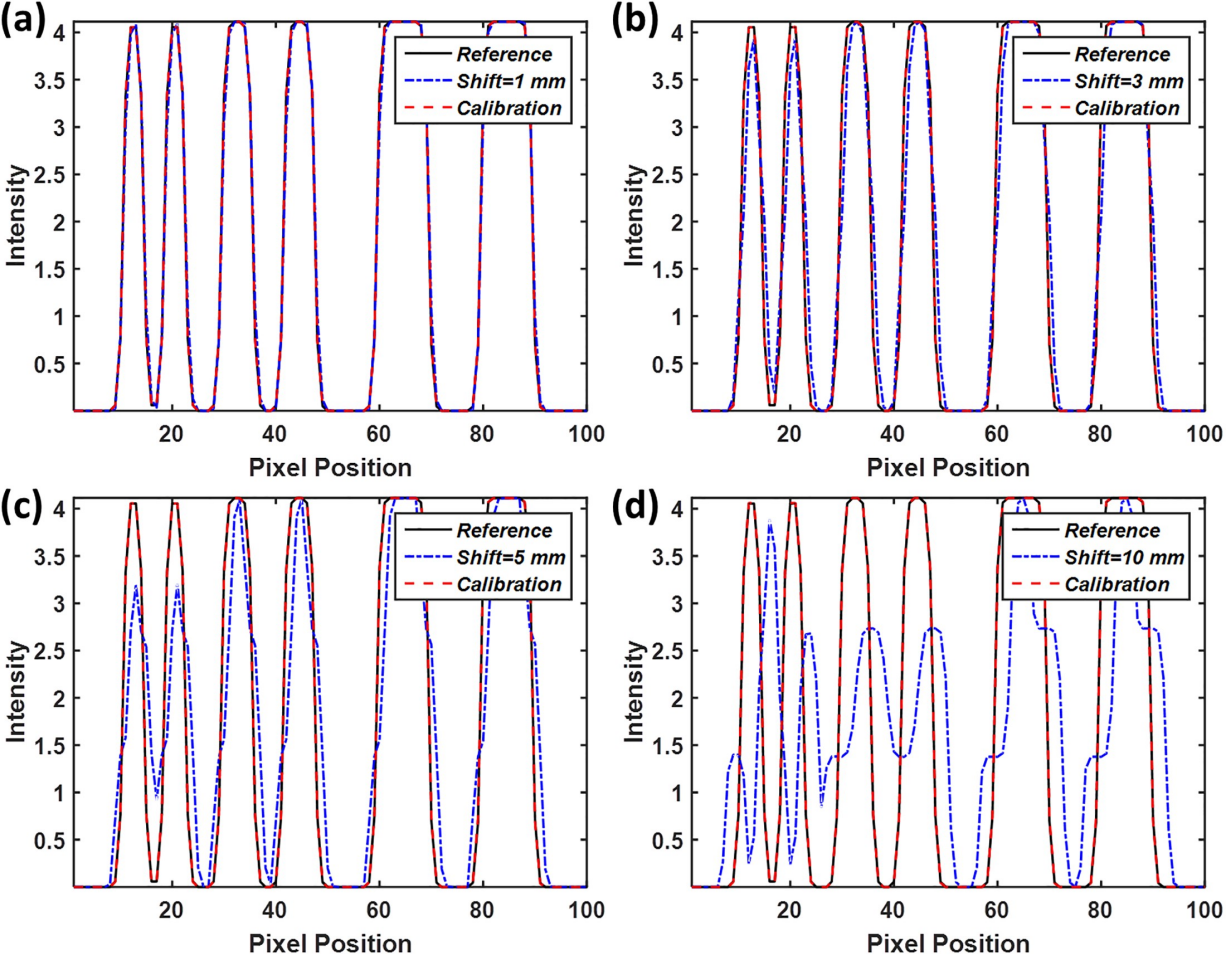
Line profiles of the simulated reconstruction images of axis shift displayed in Fig 13. Line profiles are taken in yellow line of the corresponding reference image. (a) Line profiles of the reconstructed image with and without geometric calibration when the value of axis shift is 1 mm. (b) axis shift value of 3 mm; (c) axis shift value of 5 mm; (d) axis shift value of 10 mm.

The tomosynthesis systems with various values for axis shift were simulated. The reconstructed images of the digital image quality assessment phantom are displayed in Fig 13. The images in Fig 13(a) to 13(e) were reconstructed with five different system geometries without geometric calibration, and the image artifacts were observed when increasing the value of axis shift. Fig 13(a) presents the reconstructed image with ideal system geometry and no axis shift, which can be regarded as the reference image. The reconstructed images for values in axis shift less than 3 mm were similar to the reference image; moreover, the objects in the reconstructed image for the 5-mm value in axis shift exhibited a slight offset, and the objects in the reconstructed image for the 10-mm value in axis shift demonstrated obvious offset. The images in Fig 13(f) to 13(j) were reconstructed with calibrated geometric parameters, and the offset artifacts caused by axis shift in these images were effectively suppressed. The difference images with the reference image and line profiles of the reconstructed images are presented in Figs 14 and 15, respectively, to evaluate the performance of the geometric calibration algorithm.

**Fig 16:**
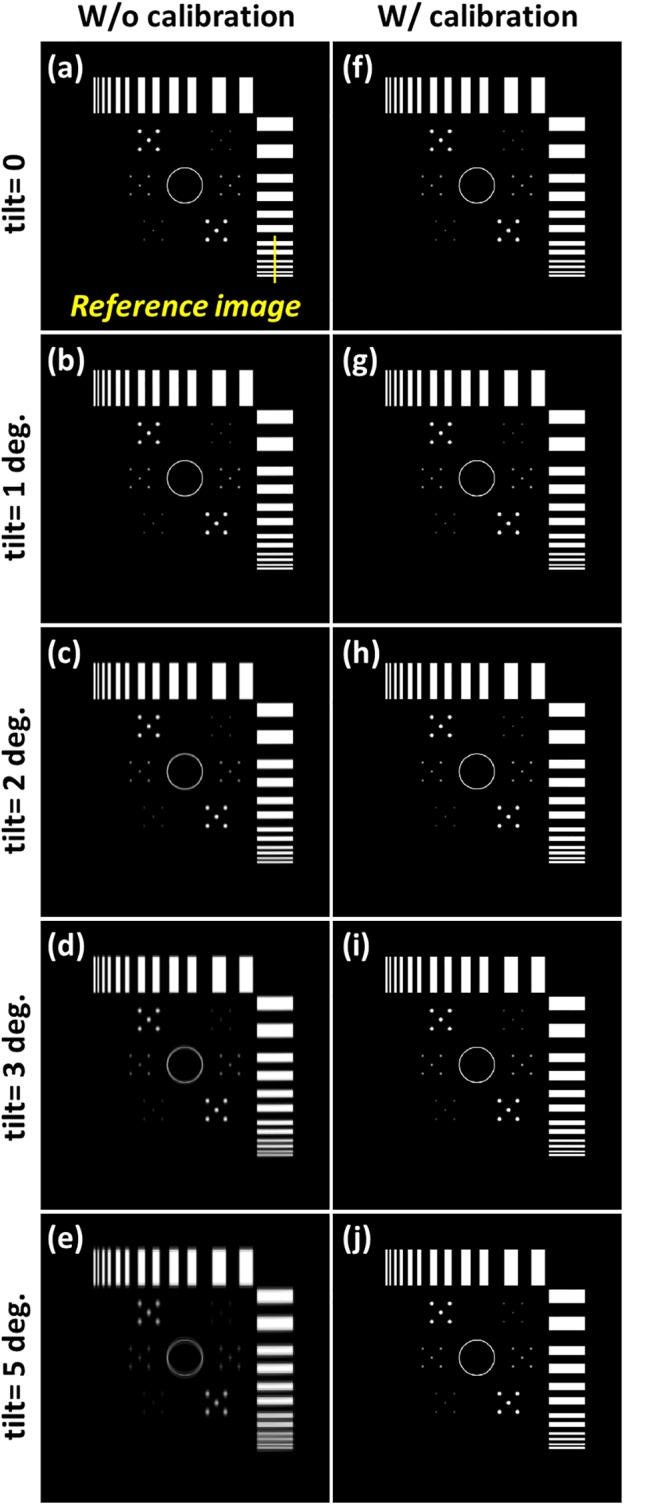
Simulated tomosynthesis reconstruction images for different axis tilt values with and without geometric calibration. (a) Reconstructed image with the ideal situation and no geometric calibration. (b) axis tilt = 1 degree; (c) axis tilt = 2 degrees; (d) axis tilt = 3 degrees; (e) axis tilt = 5 degrees. (f) Reconstructed image with the ideal situation and geometric calibration. (g) Reconstructed image of (b) after calibration. (h) Reconstructed image of (c) after calibration. (i) Reconstructed image of (d) after calibration. (j) Reconstructed image of (e) after calibration.

**Fig 17:**
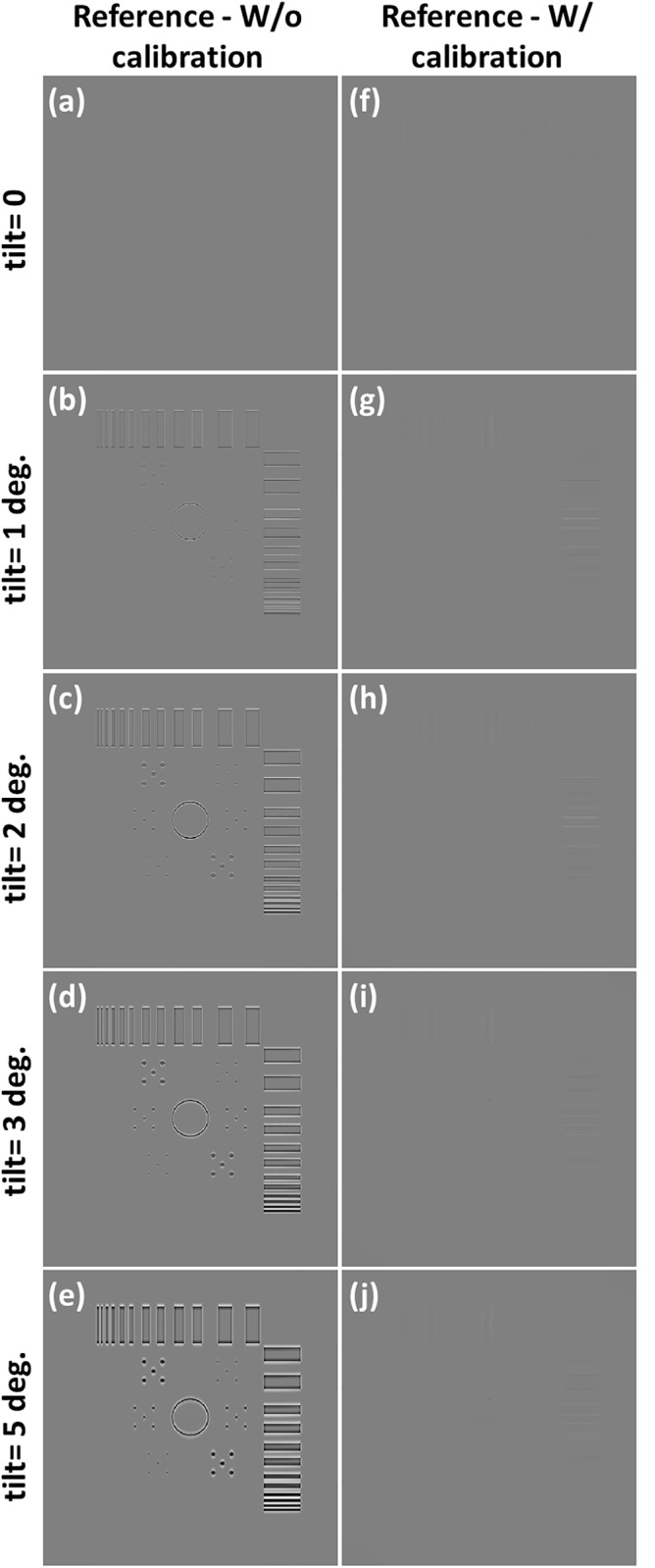
Difference images of the reference image and simulated reconstruction images of axis tilt displayed in Fig 16.

**Fig 18:**
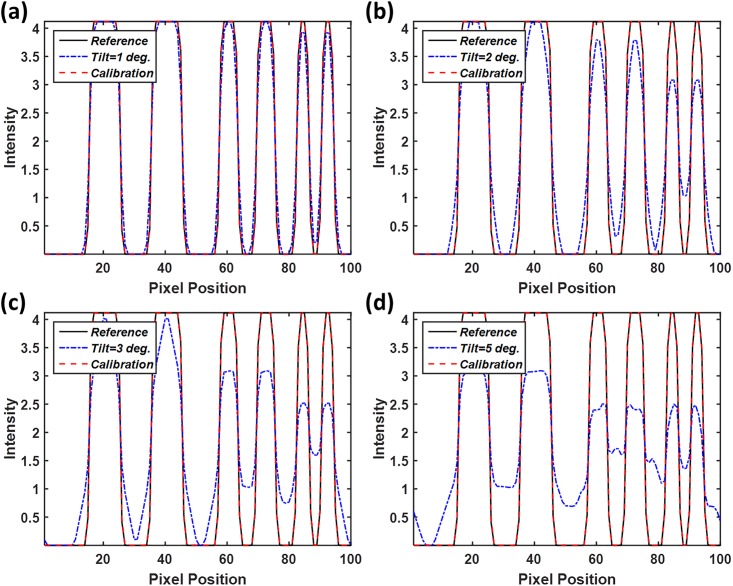
Line profiles of the simulated reconstruction images of axis tilt presented in Fig 16. Line profiles are taken in yellow line of the corresponding reference image. (a) Line profiles of the reconstructed image with and without geometric calibration when the value of axis tilt is 1 degree. (b) axis tilt value of 2 degrees; (c) axis tilt value of 3 degrees; (d) axis tilt value of 5 degrees.

The tomosynthesis systems with various axis tilt values were also simulated, as displayed in Fig 16. The images in Fig 16(a) to 16(e) were reconstructed with five different system geometries and no geometric calibration, and image artifacts were observed when increasing the value of axis tilt. Fig 16(a) presents the reconstructed image with ideal system geometry and no axis tilt, which can be regarded as the reference image. The reconstructed image for the value of axis tilt less than 1° was similar to the reference image. Furthermore, objects in the reconstructed images for axis tilt values of 2° and 3° were slightly blurred, and the objects in the reconstructed image for the value in axis shift of 5° exhibited obvious blurring. The images in Fig 16(f) to 16(j) were reconstructed with calibrated geometric parameters, and the blurring of artifacts in these images as a result of axis tilt was effectively suppressed. The difference images with the reference image and line profiles of the reconstructed images are displayed in Figs 17 and 18, respectively, to evaluate the performance of the geometric calibration algorithm.

### The experiment results of TomoDR with geometric misalignments

**Fig 19:**
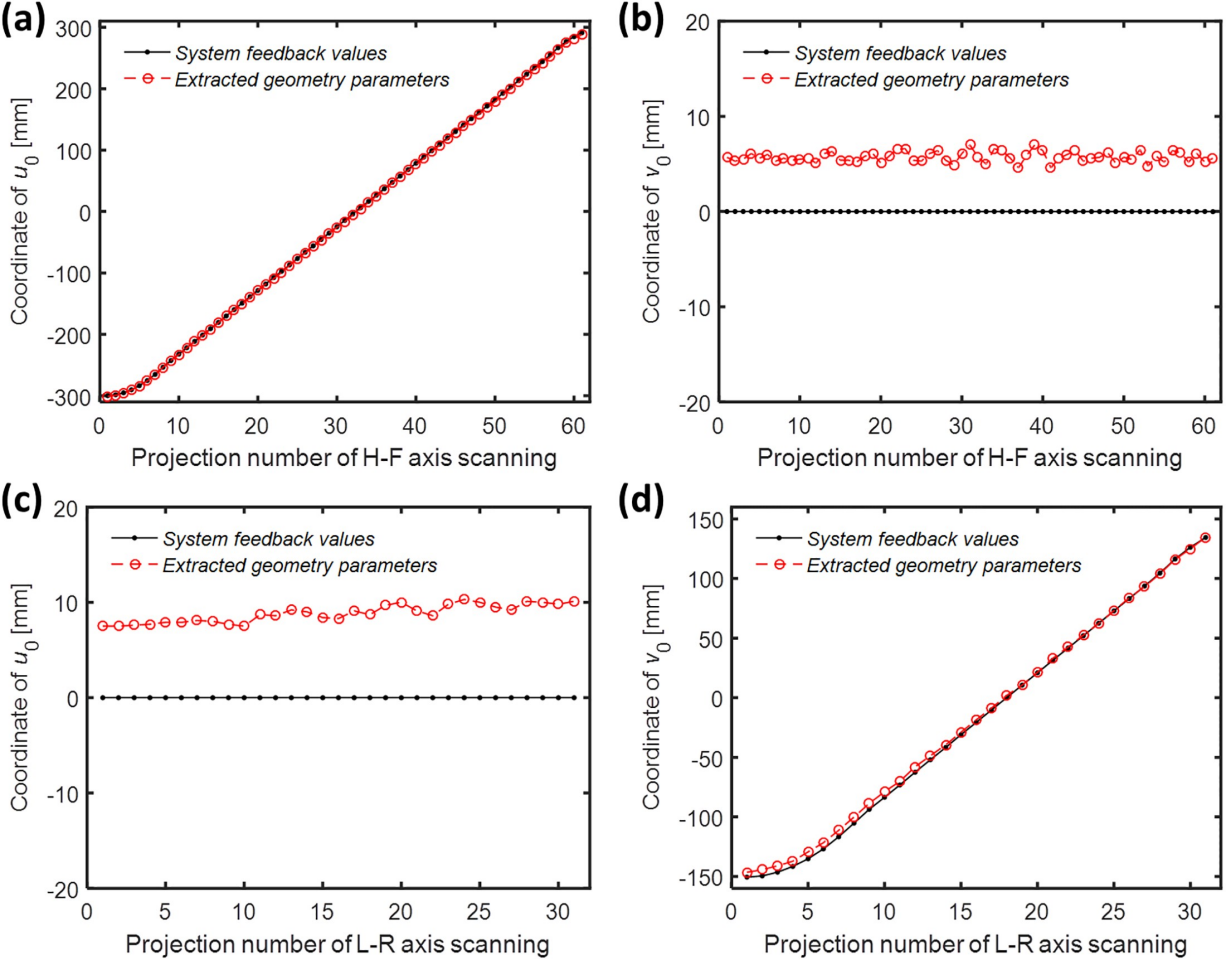
*u*_0_ and *v*_0_ coordinates of the experimental geometry extraction parameters and system feedback values. (a) *u*_0_ in HF-axis scanning. (b) *v*_0_ in HF-axis scanning. (c) *u*_0_ in LR-axis scanning. (d) *v*_0_ in LR-axis scanning.

The system alignment requirement for tomosynthesis with dual-axis scanning geometry is higher than that for single-axis scanning geometry. The relative source position of two perpendicular axes must be obtained to achieve adequate image quality for the reconstructed images. During the imaging process of TomoDR, the X-ray source moves along the HF and LR axes of the system, respectively. The height of the source and position of the image receptor are stationary during the scanning process. We therefore focused on the parameters of axis shift and axis tilt defined in accordance with changes in the offset of central rays (*u*_0_, *v*_0_) in this study. The geometric parameters were extracted using the aforementioned chest imaging protocol and geometric calibration algorithm. For the HF-axis scanning direction, the central ray offsets in the HF axis (*u*_0_) and LR axis (*v*_0_) are indicated in Fig 19(a) and 19(b), respectively. Fig 19(a) reveals that the values of the extracted parameters (red circle) were similar to those of the system embedded magnetic encoder (black dot). The extracted parameters indicated that the system demonstrated an offset of 5 to 6 mm in the LR-axis direction during scanning of the HF axis (Fig 19(b)). However, the system feedback value was zero because of the error in mechanical alignment presented in Fig 19(b) and 19(c). Fig 19(c) reveals that the system also exhibited offset of 8 to 10 mm in the HF-axis direction during scanning of the LR axis. Fig 19(d) reveals that the extracted parameters were slightly different from the system feedback values because of the LR-axis tilt. Moreover, the coordinates of the axis starting region were nonlinear increments because of the acceleration of the source position, as indicated in Fig 19(a) and 19(d).

**Fig 20:**
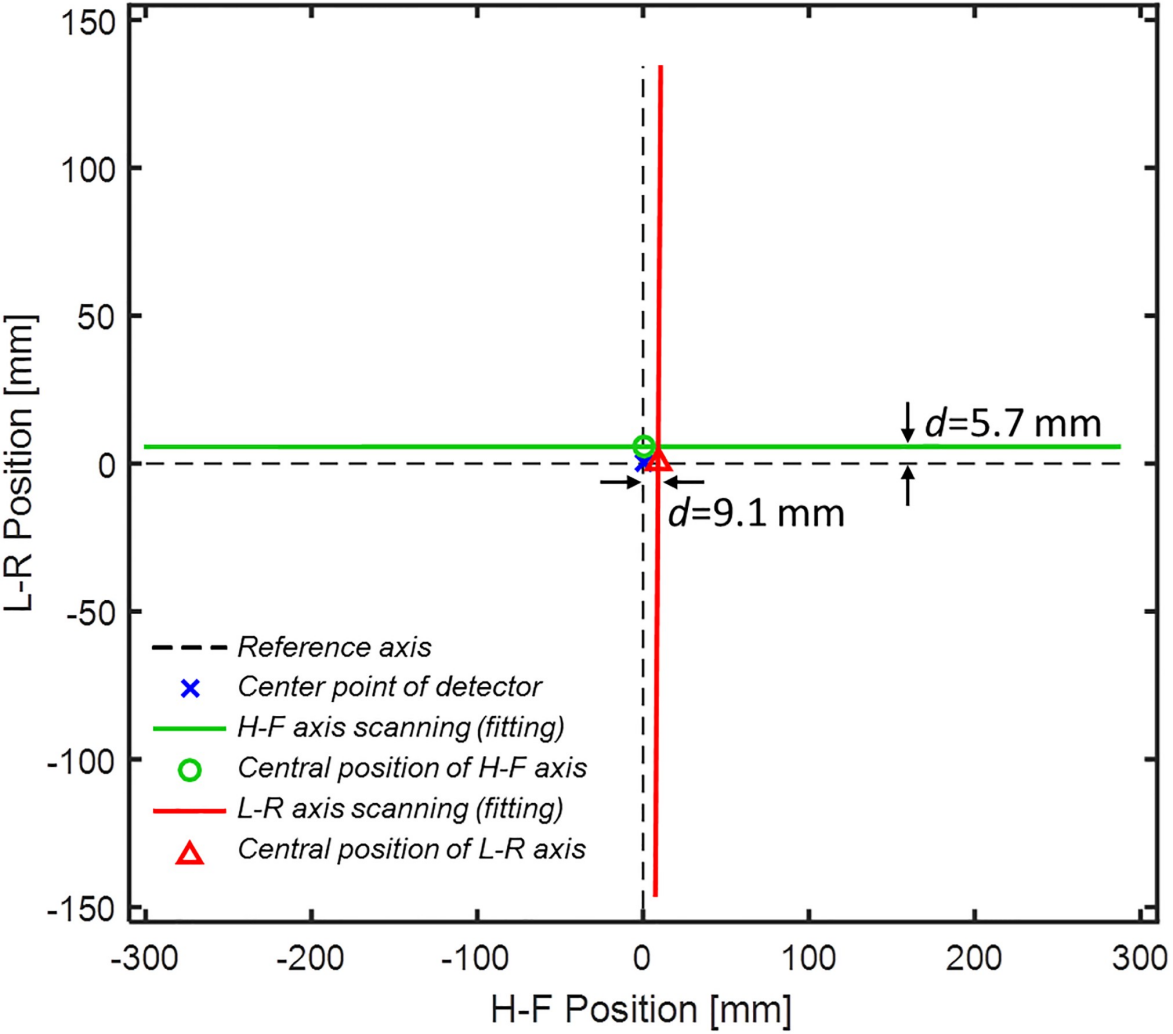
Experimental results of TomoDR with axis shift and tilt.

We used two first-order curves matching the *u*_0_ and *v*_0_ coordinates of the dual-axis scanning geometry and also indicated the central positions of these two axes (Fig 20). For the axis shift of the system, the experimental results revealed that TomoDR was the case of Fig 3(g) with shift of two axes. The LR axis exhibited an average offset of +5.7 mm in scanning of the HF axis, and the HF axis exhibited an average offset of +9.1 mm in scanning of the LR axis; furthermore, the central points of the two axes were not at the same position, indicating that the image quality must have been affected by these axis shift scenarios. In the case of system axis tilt, the angles of the HF and LR axes to the reference axis were 0.007° and 0.663°, respectively. These experimental results indicated that the HF axis was almost parallel to its reference axis. The LR axis exhibited a tilt with it reference axis, revealing that the TomoDR demonstrates single-axis tilt, similar to that displayed in Fig 4(b). However, because the angle of axis tilt was less than 1° in simulation results, the influence of axis tilt on reconstructed images was not obvious. Therefore, the influence of axis tilt is not a major factor in determining the quality of reconstructed images.

**Fig 21:**
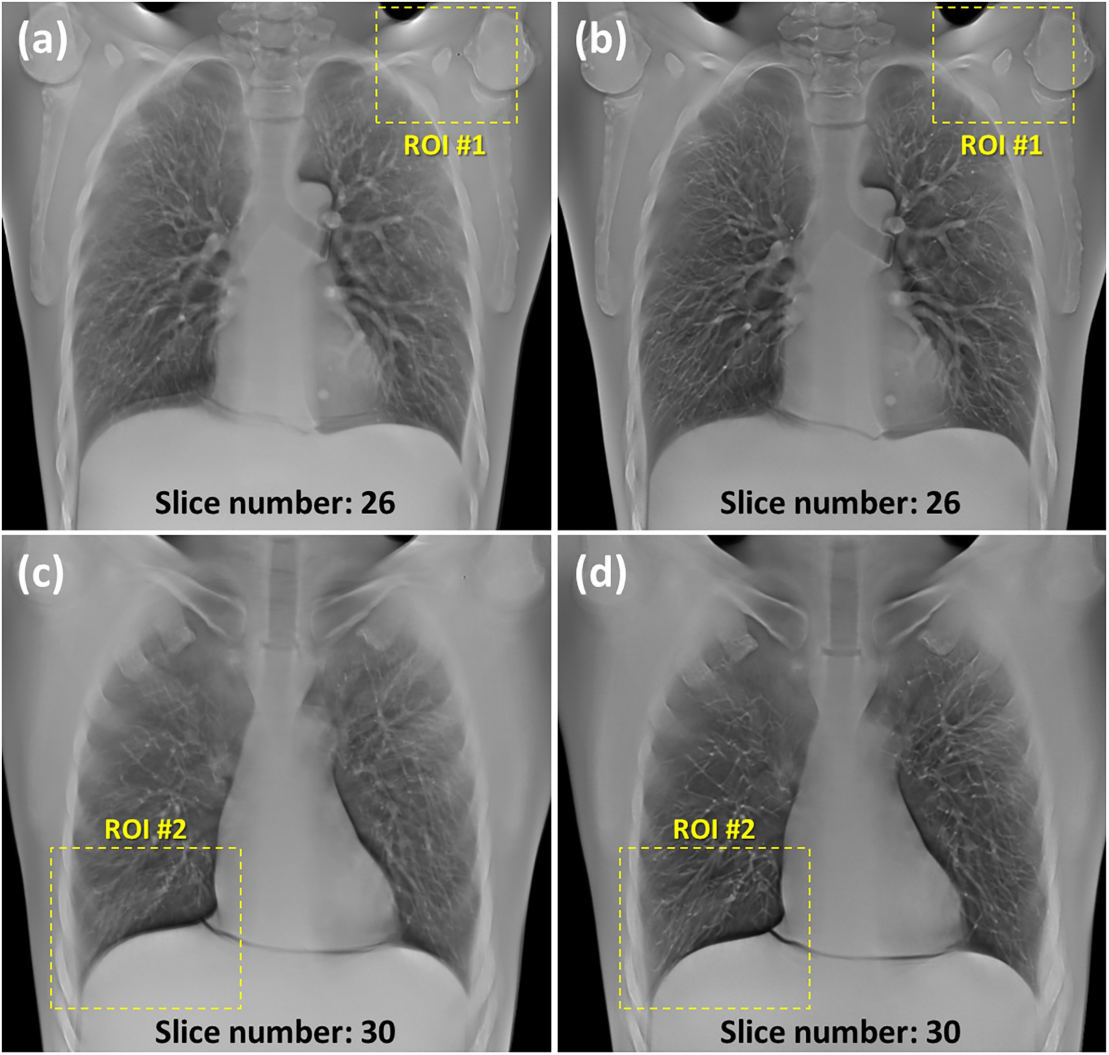
Experimental reconstruction images of anthropomorphic chest phantom of TomoDR. The phantom was scanned at 100 kV and 0.16 mAs for each projection. (a) Image of slice number 26 without geometric calibration. (b) Image of slice number 26 with geometric calibration. (c) Image of slice number 30 without geometric calibration. (d) Image of slice number 30 with geometric calibration.

**Fig 22:**
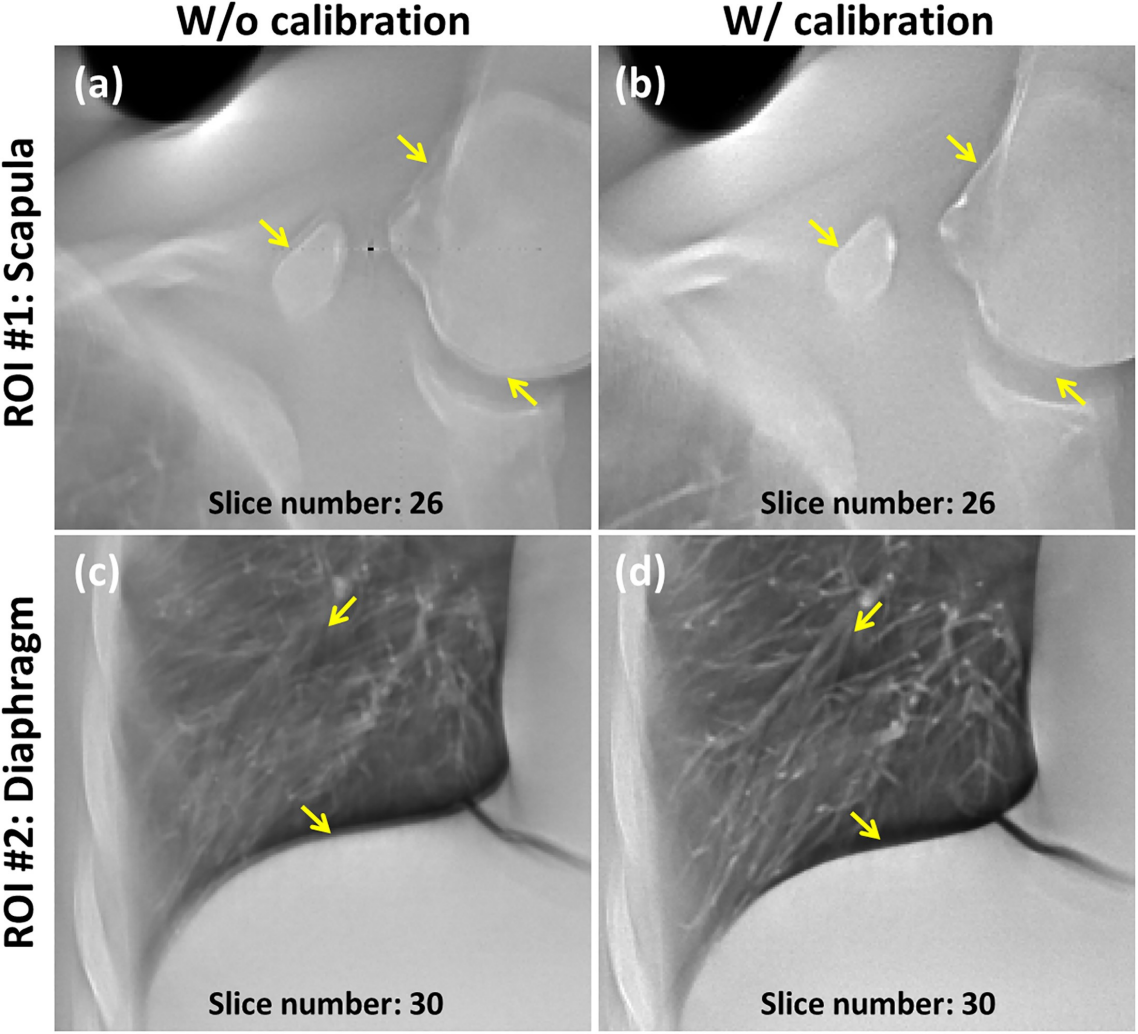
Enlarged images for the ROI of the experimental reconstruction images displayed in Fig 21. (a) Scapula area without calibration. (b) Scapula area with calibration. (c) Diaphragm area without calibration. (d) Diaphragm area with calibration.

The reconstructed images of the anthropomorphic chest phantom without geometric calibration are displayed in Fig 21(a) and 21(c), respectively. The phantom was scanned using the aforementioned chest imaging protocol. The images in Fig 21(b) and 21(d) reveal that the structures inside the chest phantom were sharper with geometric calibration. Enlarged images of the region of interest (ROI) in Fig 21 are displayed in Fig 22 for detailed comparison. Regarding the scapula area of the ROI number 1, the reconstructed images with calibration improved the image clarity and definition of the boundary between the bones and soft tissue. Regarding the area of the ROI number 2, the pulmonary blood vessels and diaphragm edge were also clearer with calibration. The experimental results of the chest phantom revealed that the geometric calibration method can help to obtain improved image quality with regard to preserving object structures and suppressing undesired artifacts. For the quantitative analysis result of the tomosynthesis reconstruction image, the MTF can be improved from 0.17 to 0.48 with the geometric calibration when the spatial frequency is 1 lp/mm. Hence, these results demonstrated the potential use of the geometric calibration for tomosynthesis with dual-axis geometry.

## Discussion and conclusions

In this study, a geometric calibration method was developed based on an iterative projection matrix based algorithm for a tomosynthesis system with dual-axis scanning geometry. To improve the calibration accuracy, we used the same scanning trajectory as the clinical chest imaging with 92 projections for the geometric calibration. The calibration time for 92 projections is less than 90 seconds. Errors of the input and extracted geometric parameters were compared based on simulated results. For the same geometry setup with the actual system, the simulated results indicated that the values for the extracted geometric parameters were similar to the input values. Additionally, a dual-axis scanning geometry tomosynthesis system with axis shift and axis tilt was simulated to evaluate the effects of geometric misalignments on the reconstructed images. The reconstructed images with and without geometric calibration were quantitatively compared. The simulated results revealed that the tomosynthesis system demonstrated a higher tolerance for geometric misalignments than did the traditional tomography system because tomosynthesis has a small magnification factor and a rotation axis proximal to the image receptor. However, the requirement of geometric alignment is higher for tomosynthesis with dual-axis scanning than for traditional tomosynthesis with single-axis scanning geometry. The simulated results also indicated that the extracted geometric parameters had excellent accuracy for tomosynthesis image reconstruction and can eliminate artifacts caused by system misalignments. Finally, to validate the performance of the geometric calibration method in experimental applications, the method was applied to the TomoDR system. After the experiment, tomosynthesis images with and without calibration were compared in different chest areas. The experimental results indicated that the method can effectively minimize artifacts and preserve object structures caused by system geometric misalignments. And the quantitative analysis result also indicated that the MTF can be further improved with the geometric calibration. In summary, the geometric calibration method can be used not only to extract geometric parameters in a tomosynthesis system but also can be applied to other tomography imaging systems to reduce the occurrence of geometric misalignments. Furthermore, this method can also be used for other phantom configuration with known 3D marker coordinates.
